# The assembly of stress granules during foot-and-mouth disease virus infection is uncoupled from activation of cellular intrinsic antiviral signalling

**DOI:** 10.1371/journal.ppat.1013722

**Published:** 2026-06-11

**Authors:** Mariana Marques, Yui Xi Wong, Zhaozhi Sun, Amina Yasmin, J. Paul Taylor, Alessia Ruggieri, Tobias J. Tuthill, Nicolas Locker

**Affiliations:** 1 The Pirbright Institute, Pirbright, Surrey, United Kingdom; 2 The University of Manchester, Manchester, United Kingdom; 3 Heidelberg University, Medical Faculty Heidelberg, Department of Infectious Diseases, Molecular Virology, Centre for Integrative Infectious Disease Research, Heidelberg, Germany; 4 Department of Cell and Molecular Biology, St. Jude Children’s Research Hospital, Memphis, Tennessee, United States of America; Karolinska Institutet, SWEDEN

## Abstract

Foot-and-mouth disease virus (FMDV) is highly contagious among cloven-hoofed animals and poses a major threat to the livestock industry worldwide. A fundamental gap in knowledge for high consequence viruses such as FMDV is understanding how the virus evolved to evade cellular antiviral responses. FMDV belongs to the *Picornaviridae*, a family of positive-sense single-stranded RNA viruses. The detection of viral double-stranded viral RNA intermediates during infection can trigger both the assembly of cytoplasmic stress granules (SGs) and the activation of the RIG-I-like receptors (RLR)-mediated innate immune response (IIR). FMDV has been proposed to antagonize these mechanisms, suggesting that both can limit viral replication. In this study, we investigate the dynamic and importance of SG assembly for IIR activation upon dsRNA stimulation or FMDV replication in porcine epithelial kidney cells. First, we show that the formation of SG following a challenge with poly(I:C), a viral dsRNA mimic, does not modulate the activation of IIR. Our data further reveal transient assembly of SG during FMDV infection followed by virus-induced cleavage of G3BP1, a core SG protein. While SG assembly does not impact viral replication or antiviral response activation, we demonstrate that preventing their disassembly negatively impacts FMDV replication. Overall, we show that SGs assembly during infection does not modulate viral replication and is uncoupled from IIR activation, while FMDV actively cleaves G3BP1 by a 3C^pro^-mediated mechanism to promote their disassembly, suggesting a potential antiviral role for persistent SGs.

## Author summary

Biomolecular condensates, including stress granules, are key regulatory compartments that control fundamental cellular processes. By condensing RNAs and proteins, these structures enable cells to rapidly adapt to stress, such as viral infection and importantly the cell-autonomous innate immune response is proposed to be regulated by biomolecular condensates. However, the molecular mechanisms underpinning these functions remain unclear and controversial. To date, most studies have relied on the use of genetic knock out that do not enable to disentangle the roles of condensate proteins dependent or independent from their location. We used specific condensation inhibitor to circumvent these issues and dissect the interplay between stress granules and the innate response upon the stimulation with a viral dsRNA mimic or foot-and-mouth disease virus infection. We demonstrate that infection triggers the formation of stress granules independently from activation of innate signalling pathways. We also show that stress granules persistence attenuates viral replication posing them as part of the cell’s response that viruses must overcome or subvert to replicate.

## Introduction

Foot-and-mouth disease virus (FMDV) is the aetiological agent of foot-and-mouth disease (FMD), a highly contagious diseases that infects a broad range of wild and domestic cloven-hoofed animals [1]. FMD is endemic in Africa and Asia, where it is continuously controlled by vaccination, yet outbreaks in FMD-free regions, as recently in Europe, require strict restrictions [2]. Causing severe pain and distress in the infected animals, FMD seriously affect livestock and its industry worldwide, threatening the international trade in animals and animal products with severe economic implications [3].

FMDV belongs to the family *Picornaviridae* of non-enveloped viruses with single-stranded positive-sense RNA genome, which includes a long 5′-untranslated region (5′UTR), a large open reading frame (ORF), and a short 3′UTR. Translation begins at the internal ribosome entry site (IRES) element in the 5’UTR to produce a polyprotein encoding for eight non-structural proteins that regulate RNA replication, protein folding and virus assembly, and four structural proteins, constituting the capsid [4].

During RNA virus infection, the detection of double-stranded RNA (dsRNA) replication intermediates or deregulation of cellular homeostasis activates the integrated stress response (ISR), which results in an adaptive transcriptional and translational rewiring to limit viral protein synthesis and promote cellular recovery [5]. This process is mediated by the phosphorylation of the eukaryotic translation initiation factor 2α (eIF2α) and involves polysome disassembly and the consequent accumulation of stalled mRNAs, which serve as scaffold to nucleate RNA-binding proteins (RBPs), such as Ras-GTPase activating protein-binding protein 1 (G3BP1). This triggers the recruitment and accumulation of proteins into ribonucleoprotein complexes that ultimately mature into cytoplasmic membrane-less condensates, named stress granules (SGs), by liquid-liquid phase separation [6–10]. SGs are highly heterogeneous and dynamic, rapidly assembling in response to stress and dissolving upon its resolution to resume the translation of stalled mRNAs [11]. Several studies have identified and characterized SG-like or related condensates during infection, including paracrine granules and RNAse-L bodies (RLBs) that differ from canonical SGs in terms of composition and function [5,12,13]. These observations highlight the diverse nature of stress-induced biocondensates, and the importance of context and composition for their biological function.

The exact function of SGs and SG-like condensates during viral infection and their interplay with the cell intrinsic antiviral innate immune response (IIR) is unclear. The detection of dsRNA intermediates triggers an IIR mediated by cytoplasmic RIG-I-like receptors (RLR) [14], relaying a signalling cascade mediated by mitochondrial antiviral signalling (MAVS) to trigger an interferon (IFN)-mediated transcriptional induction of antiviral genes [15]. SGs have been proposed to function as either antiviral platforms or signalling scaffolds for RLRs or other antiviral effectors [16–22]. In turn, numerous viruses have evolved strategies to antagonize SG formation or promote their disassembly [5]. SGs have also been described as “shock absorbers” that dampen antiviral signalling by sequestering antiviral factors and preventing or delaying their activation to protect cells from immune-mediated apoptosis [23]. Alternatively, SGs could be simple by-products of viral infections and independent from antiviral immune responses [12,18,24].

FMDV has been previously shown to interfere with host translation and SG components to evade these mechanisms. Both FMDV proteases, L^pro^ and 3C^pro^, are associated with cleavage of G3BP1 to antagonize SGs, yet whether SGs assemble during infection or their impact on IFN signalling remains unclear [25,26].

Herein, we investigated the significance of SGs during RNA virus infection in porcine epithelial kidney cells (PK-15) and explored their relevance for the activation of the intrinsic IIR. First, we show that the induction of IIR signalling and the assembly of G3BP1-foci are uncoupled following the stimulation with poly(I:C), a synthetic viral dsRNA mimic. We also demonstrate that stimulation of the RIG-I/MAVS pathway is not sufficient to induce the formation of G3BP1-foci. Furthermore, our data show that FMDV infection promotes SGs assembly followed by their rapid disassembly and cytopathic effect. Inhibiting G3BP1 condensation and foci assembly does not impact FMDV replication, neither modulates the IIR against a mutated FMDV that lacks the ability to evade IFN signalling. Lastly, our data indicate that delaying the disassembly of FMDV-induced G3BP1-foci during infection reduces FMDV replication. Overall, this sheds light on the interplay between of biocondensates dynamics and IIR signalling during infection.

## Results

### Inhibiting G3BP1 condensation blocks poly(I:C)-induced foci, but does not revert IIR signalling

PK-15 are porcine kidney epithelial cells commonly used to study FMDV infection. To ensure appropriate monitoring of ISR and IIR induction, we established that this cell line is responsive to synthetic viral dsRNA mimics, such as 3p-hpRNA and poly(I:C). 3p-hpRNA is a specific RIG-I ligand, consisting of a short RNA hairpin structure with an uncapped 5’ triphosphate extremity [27], whereas poly(I:C) is recognized by several cytosolic receptors, including RIG-I-like receptors (RLR) [28], protein kinase R (PKR) [12] and oligoadenylate synthetase-like (OAS) [29]. To characterize the antiviral response initiated by 3p-hpRNA and poly(I:C) in PK-15, cells were transfected for 6 hours with increasing amounts of each viral mimic prior to measuring the levels of IIR and ISR factors using RT-qPCR or western blot (**S1A-B** Fig).

As expected, increasing amounts of 3p-hpRNA resulted in a dose-dependent induction of *IFNB1* and ISGs expression, including *ISG15*, *ISG56*, *IRF1*, *IL6*, *and EIF2AK2* (coding for PKR), as seen by qPCR (**S1A** Fig). Similarly, we detected higher levels of IRF3 and STAT1 phosphorylation and ISGs, such as IFIT2, IRF1, ISG15 using immunoblotting (**S1B** Fig). Transfection with increasing amounts of poly(I:C) promoted a stronger *IFNB1* and ISGs expression (**S1A** Fig), accompanied by higher levels of IRF3 phosphorylation (**S1B** Fig), but a plateau or decrease in the phosphorylation of STAT1 and ISGs synthesis (IFIT2, IRF1, ISG15) (**S1B** Fig). This could be explained by the ISR activation and resulting decrease in translation following poly(I:C) but not 3p-hpRNA stimulation (**S1B** Fig). In summary, these data support the use of viral mimics to induce antiviral response in our model system.

**Fig 1:**
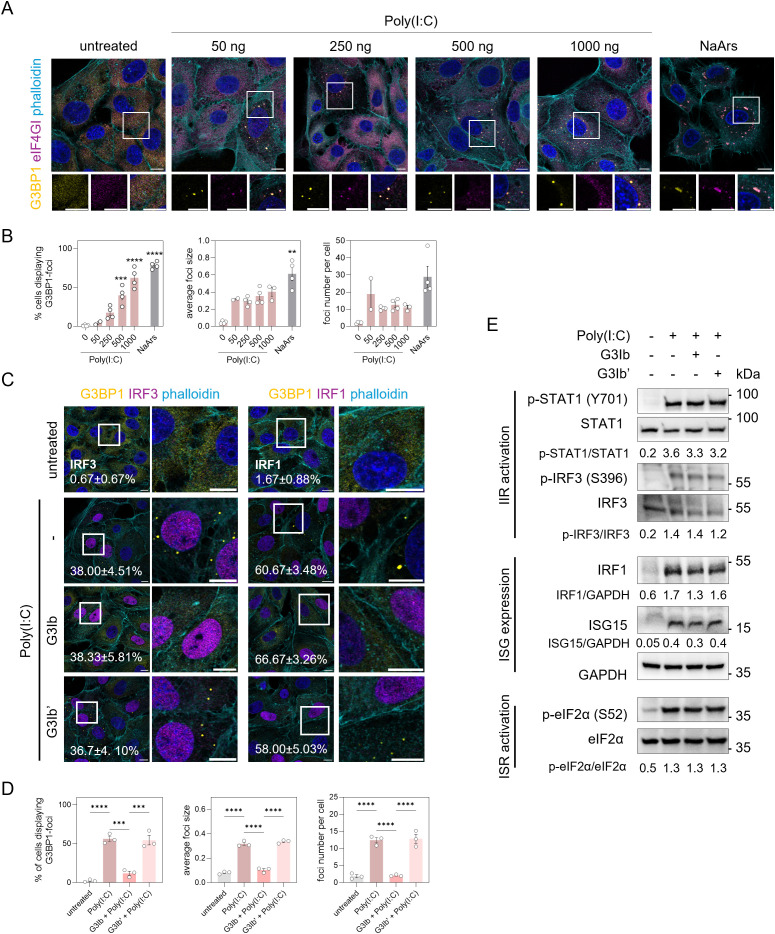
Inhibiting G3BP1 condensation blocks poly(I:C)-induced foci. **(A)** PK-15 cells transfected with increasing concentrations of poly(I:C) for 6 hours. Sodium arsenite treatment (1 mM for 1 hour) was used as positive control. Cells were analysed by immunofluorescence for the SG markers G3BP1 (gold) and eIF4GI (magenta), and a F-actin marker phalloidin (cyan). Nuclei were stained with DAPI. Scale bars represent 10 μm. **(B)** The percentage of cells displaying G3BP1-foci was quantified by manual counting of at least 100 cells per replicate. The average size of foci per cell and the number of foci per cell were quantified using the G3BP1 channel and ‘Analyse Particles’ plugin from ImageJ. Results shown as mean ± SEM, n=3, representing the average of at least 30 cells per condition. **p<0.01, *** p<0.001, ****p<0.0001; using ordinary one-way ANOVA with Šidák’s multiple comparison post-test to 50 ng poly(I:C). **(C)** PK-15 cells transfected with poly(I:C) for 6 hours following treatment with G3BP1 condensation inhibitor (G3Ib) or its enactive enantiomer (G3Ib’). Cells were analysed by immunofluorescence for the SG markers G3BP1 (gold), innate immune response activation markers IRF3 or IRF1 (magenta), and a F-actin marker phalloidin (cyan). Nuclei were stained with DAPI. Scale bars represent 10 μm. The percentage of cells that show nuclear IRF3 or IRF1 is showing on the images as mean ± SEM. **(D)** Quantification done as in **(B)**. *** p < 0.001, ****p < 0.0001; using ordinary one-way ANOVA with Šidák’s multiple comparison post-test to untreated. **(E)** Representative western blot from at least three independent experiments for markers of interferon signalling activation (STAT1 and IRF3 phosphorylation levels), interferon-stimulated genes expression (IRF1 and ISG15), and integrated stress response activation (phosphorylation of eIF2α). Molecular weights are indicated on the right. Band intensities normalised to total protein or GAPDH.

Next, we set out to dissect the assembly of SGs in PK-15 cells following a 6-hour treatment with increasing amounts of poly(I:C) or 3p-hpRNA. Sodium arsenite was used as a positive control for the assembly of canonical eIF2α-dependent SGs [30]. Cells were stained using the SG markers G3BP1 and eIF4GI (Figs 1A-1B and **S1C**). Arsenite treatment resulted in accumulation of G3BP1 and eIF4GI into foci in 78.50 ± 2.33% of the cells, reflecting SG assembly, with an average number of 28.92 ± 6.16 foci per cell with an average size of 0.61 ± 0.08 μm^2^. Adding increasing amounts of poly(I:C) resulted in the dose-dependent induction of foci, in similar number and size in all the tested conditions (18.91 ± 9.09, 10.47 ± 0.48, 12.53 ± 1.68 and 10.99 ± 1.12, and 0.32 ± 0.01, 0.29 ± 0.03, 0.35 ± 0.05 and 0.40 ± 0.05 μm^2^, respectively), but less numerous and smaller in size when compared to canonical arsenite-induced SGs (Fig 1A-1B). In contrast, 3p-hpRNA did not induced the assembly of G3BP1-foci (**S1C** Fig). This suggests that the assembly of G3BP1-foci is triggered independently of the RIG-I/MAVS-mediated response and therefore relies on the poly(I:C) stimulation of other receptors, including PKR or OASs.

To address the dynamics of ISR and IIR activation following poly(I:C) stimulation, we conducted a time-course experiment from 1 to 6 hours post-transfection (hpt) and monitored both the assembly of G3BP1-foci and IIR activation using immunofluorescence assays, using G3BP1 as SG/ISR marker, or IRF3 and IRF1 as marker of IIR signalling (**S2A-C** Fig). Upon RIG-I/MAVS signalling, the activation of IRF3 results in its intrinsic nuclear translocation, followed by an interferon-mediated expression of ISG in both autocrine and paracrine ways. The nuclear localization of IRF1 was used as measure of ISG expression, as this feature is lost upon inhibiting interferon-mediated signalling with ruxolitinib (**S2D** Fig).

Following poly(I:C) treatment, G3BP1-foci assembled in 4.67 ± 0.88% of the cells from 1 hpt, reaching 45.00 ± 2.00% of the cells at 6 hpt. The number of foci per cell was higher at 1 hpt (38.5 ± 3.8) compared to the later time points (17.9 ± 1.9, 13.4 ± 0.7, 11.6 ± 0.8 and 11.6 ± 1.6 for 2, 4, 6 and 8 hpt, respectively), whereas their size at 1 hpt was smaller (0.31 ± 0.05 μm^2^) compared to later times (0.40 ± 0.06, 0.44 ± 0.03, 0.40 ± 0.02 and 0.43 ± 0.02 μm^2^) (**S2B** Fig). This may reflect the proposed multistep process for SGs assembly whereby small aggregates coalesce before fusing into larger condensates [31].

Our analysis revealed that the activation of the RIG-I/MAVS/IRF3 pathway and the assembly of G3BP1-foci occur within the same cell, as reflected by the intensity levels of nuclear IRF3 in cells with and without foci (**S2C** Fig). Interestingly, the nuclear localization of IRF1 was not detected in cells assembling G3BP1-foci upon poly(I:C) transfection (**S2C** Fig), suggesting these cells are unable to efficiently produce antiviral factors. Overall, this suggests that IIR and ISR activation occur at a similar time, raising the question of whether one drives the other, and suggests that ISR activation might impair the IFN-mediated response.

To establish whether the ability to assemble SGs would impact IIR induction, we leveraged a pharmacological inhibitor of SG assembly, the G3BP condensation inhibitor b (G3Ib), which binds the G3BP1 domain essential for the conformational change driving SG assembly and the nucleation of other SG proteins [32], being G3Ib’ its inactive enantiomer used as control. G3Ib, but not G3Ib’, prevents the formation of condensates containing G3BP1, PABP, Caprin1, G3BP2 and eIF4GI, following sodium arsenite treatment (**S2E** Fig). Therefore, this compound allows to discriminate the role of SGs from that of their individual components, presenting an advantage over G3BP1/2 knocked out genetic models, which does not discriminate between loss of G3BP1/2 SG-dependent functions and SG-independent functions, such as modulating the innate immune response or RNA metabolism [19,21].

We then tested the ability of G3Ib to prevent the assembly of poly(I:C)-induced SGs and their relevance for IIR activation. To visualize G3BP1-foci assembly and IIR activation, cells were labelled for G3BP1 and IRF3 or IRF1, respectively (Fig 1C). Treating cells with G3Ib resulted in impaired foci assembly following poly(I:C) compared to untreated cells, with reduction in percentage of cells with G3BP1-foci from 56.33 ± 3.53% to 12.00 ± 2.65%, while the inactive drug G3Ib’ had no impact (Fig 1D). The number of G3BP1-foci per cell and average size were also reduced in cells treated with G3Ib (2.08 ± 0.16 and 0.10 ± 0.01 μm^2^) compared to poly(I:C) treatment alone (12.41 ± 0.79 and 0.32 ± 0.01 μm^2^), while G3Ib’ had no significant effect (12.82 ± 1.34 and 0.34 ± 0.01 μm^2^) (Fig 1D). This supports the use of G3Ib as a tool to prevent the assembly of poly(I:C)-induced G3BP1-foci and characterise their functions during IIR activation.

We further quantified the percentage of cells displaying IIR activation following poly(I:C) transfection upon G3Ib or G3Ib’ treatments. Triggering the IIR pathway resulted in IRF3 nuclear translocation upon poly(I:C) stimulation (38.00 ± 4.51%), compared to non-transfected cells (0.67 ± 0.67%). Preventing the formation of G3BP1-foci with G3Ib or the inactive G3Ib’ did does not alter IRF3 nuclear translocation (38.33 ± 5.81% and 36.67 ± 04.10%) (Fig 1C). Likewise, IRF1 expression increased upon poly(I:C) stimulus (60.67 ± 3.48%) compared to untreated samples (1.67 ± 0.88%), while no significant changes occurred upon G3Ib or G3Ib’ treatment (66.67 ± 3.23% and 58.00 ± 5.03%) (Fig 1C).

These observations are consistent with the unaltered expression of *IFNB1* or ISGs (*ISG56*, *IRF1*, *ISG15*, *IL6*, and *EIF2AK2*) at the mRNA level (S2F Fig), or the unchanged levels of IIR-related factors, including IRF3 and STAT1 phosphorylation, as well as ISGs expression (IRF1 and ISG15) (Fig 1E). The activation of the ISR, monitored through levels of eIF2α phosphorylation was also unaffected by G3Ib or G3Ib’ treatment in poly(I:C)-stimulated samples (Fig 1E). Overall, this suggests that the assembly of the poly(I:C)-induced SGs is uncoupled from the induction of intrinsic IIR signalling following viral mimic challenge.

### FMDV infection induces the assembly of G3BP1-foci

Having shown that the assembly of G3BP1-foci does not impact IIR signalling in response to viral mimics, we addressed the assembly and dynamics of biocondensates formation upon infection of PK-15 cells with FMDV. Previous studies have suggested that FMDV disrupts SG formation, however conflicting data exist regarding the mechanisms involved [25,26].

**Fig 2:**
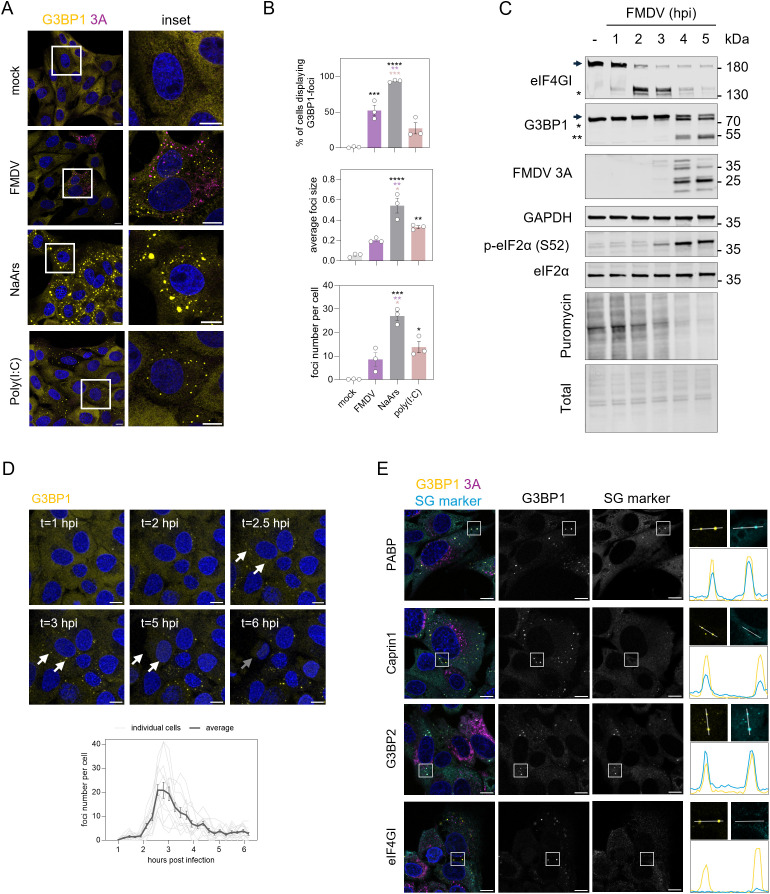
FMDV induces the formation of transient G3BP1-foci during infection. **(A-B)** PK-15 cells were infected with FMDV for 3.5 hours. Sodium arsenite treatment (1 mM, 1 hour) and poly(I:C) transfection (500 ng) were used as positive controls. Cells were analysed by immunofluorescence for the SG markers G3BP1 (gold) and FMDV 3A (magenta). Nuclei were stained with DAPI. Scale bars represent 10 μm. The percentage of cells displaying G3BP1-foci was quantified by manual counting of cells of at least 100 cells per replicate. The average size of foci per cell and the number of foci per cell were quantified using the G3BP1 channel and ‘Analyse Particles’ plugin from ImageJ. Results shown as mean ± SEM of at least three independent biological replicates, representing the average of at least 30 cells per condition. *p < 0.05; **p < 0.01; ***p < 0.001; ****p < 0.0001; using ordinary one-way ANOVA with Šidák’s multiple comparison post-test. **(C)** Representative western blot from at least three independent experiments for eIF4GI and G3BP1 cleavage, viral infection (FMDV 3A), and integrated stress response activation (phosphorylation of eIF2α and puromycin). Molecular weights are indicated on the right. **(D)** PK-15 G3BP1-RFP were infected with FMDV and monitored by live-cell imaging for up to 7 hpi. White arrows indicate SGs being formed and disappearing in an infected cell. Grey arrow indicates the same cell dying. The number of SG per cell of 13 cells that died during this time, reflecting a full cycle of infection, were quantified using the G3BP1 channel and ‘Analyse Particles’ plugin from ImageJ. **(E)** Cells were analysed by immunofluorescence for the SG markers G3BP1 (gold) and FMDV 3A (magenta), as well as a second SG marker as referred. Nuclei were stained with DAPI. Scale bars represent 10 μm. Colocalization plots show the distribution of fluorescence intensity (y -axis, arbitrary units) across the white line (x-axis, distance in microns) of G3BP1 (gold) and the second SG marker (cyan).

PK-15 cells were infected with FMDV for 3.5 hours and the assembly of G3BP1-foci and FMDV infection monitored using immunofluorescence labelling for G3BP1 and the viral 3A protein, respectively. FMDV infection resulted in the assembly of G3BP1-foci in 52.33 ± 7.31% of the infected cells at this point (Fig 2A-B), compared to 93.67 ± 0.88% and 27.67 ± 7.69% in cells treated with the positive controls sodium arsenite or poly(I:C), respectively. FMDV-induced G3BP1-foci were present in lower numbers (8.56 ± 2.90) and smaller in size (0.20 ± 0.01 μm^2^) when compared to those induced by sodium arsenite (27.03 ± 1.93, 0.54 ± 0.07 μm^2^) and poly(I:C) (13.87 ± 2.38, 0.33 ± 0.01 μm^2^).

FMDV infection results in translational shut-off in infected cells, with evidence of both eIF2α-dependent and independent shut-off signalling [26,33,34]. To better understand the dynamics of protein synthesis during FMDV infection in PK15 cells, we monitored translation using puromycylation assays and detecting eIF4GI integrity, whose cleavage has been associated with host shut-off during picornavirus infection [33,35,36]. FMDV life cycle is rapid with a considerable cytopathic effect (CPE) observed after 6 hpi, therefore these analyses were carried out hourly up to 5 hpi (Fig 2C). Infection was monitored by detecting the non-structural viral protein 3A and its polyprotein precursors. Cleavage of eIF4GI was detected as early as 1 hpi, followed by an increase in the eIF2α phosphorylation levels and decrease in translation rates, measured by reduced puromycin intensity, starting at 3 hpi. In addition, G3BP1 cleavage was detected from 4 hpi onwards.

To follow the kinetics of G3BP1-foci assembly during FMDV infection, we engineered PK-15 cells expressing endogenous RFP-tagged G3BP1, using CRISPR-Cas9-based gene editing. First, we confirmed that these cells respond to FMDV infection similarly to parental PK-15 cells, displaying similar patterns of eIF4GI and G3BP1 cleavage, eIF2α phosphorylation and translational shut off, as well a similar viral replication kinetics (S3A-B Fig). Using this system, we followed the assembly and disassembly of G3BP1-RFP foci in cells with visible CPE up to 6 hpi. Foci assembly peaked at around 3 hpi (20.77 ± 3.37 foci number per cell) followed by their disassembly and appearance of CPE (Fig 2D). These observations confirm that G3BP1-foci are induced early during FMDV infection and disassemble at later times points, when we observe G3BP1 cleavage.

To better characterize the FMDV-induced condensates, we further performed colocalization assays between G3BP1 and other canonical SG markers, including PABP, Caprin1, G3BP2 and eIF4G, upon infection (Fig 2E). All markers, except eIF4GI, colocalize with G3BP1 in SGs during infection with FMDV, suggesting that FMDV-induced granules share components with previously described canonical SGs.

### Preventing G3BP1 condensation has no effect on FMDV infection or propagation

**Fig 3:**
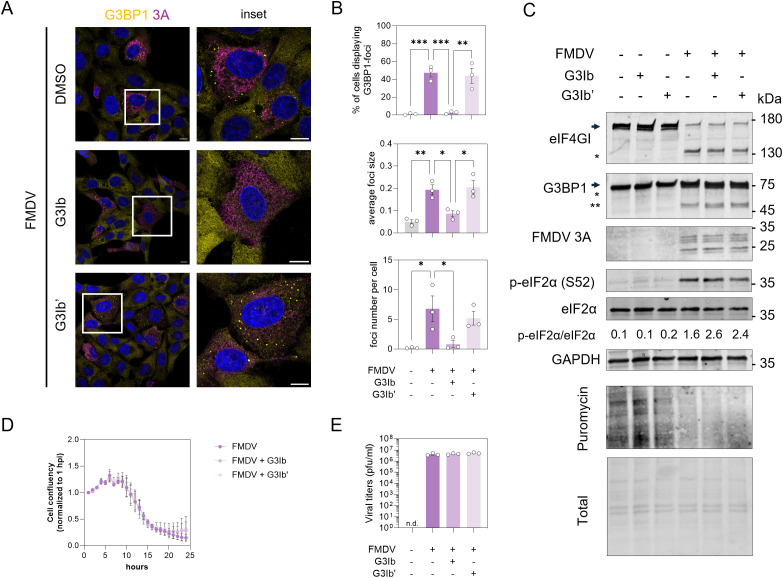
Preventing G3BP1 condensation does not interfere with FMDV replication. **(A-C)** PK-15 cells were infected with FMDV for 3.5 hours following treatment with G3BP1 condensation inhibitor (G3Ib) or its enactive enantiomer (G3Ib’). **(A)** Cells were analysed by immunofluorescence for the SG markers G3BP1 (gold) and FMDV 3A (magenta). Nuclei were stained with DAPI. Scale bars represent 10 μm. **(B)** The percentage of cells displaying G3BP1-foci was quantified by manual counting of at least 100 cells per replicate. The average size of foci per cell and the number of foci per cell were quantified using the G3BP1 channel and ‘Analyse Particles’ plugin from ImageJ. Results shown as mean ± SEM of at least three independent biological replicates, representing the average of at least 30 cells per condition. *p<0.05; **p<0.01; ***p<0.001; using ordinary one-way ANOVA with Šidák’s multiple comparison post-test. **(C)** Representative western blot from at least three independent experiments for eIF4GI and G3BP1 cleavage, viral infection (FMDV 3A), and integrated stress response activation (phosphorylation of eIF2α and puromycin). Molecular weights are indicated on the right. **(D)** PK-15 cells were infected with FMDV at a MOI of 0.01 in the presence or absence of G3Ib or G3Ib’, alongside a mock-infected controls, and cell confluence was monitored every hour for 24 hours, using an IncuCyte Zoom. Data represents an average of three replicates, normalized to the first time-point measured (1 hpi). **(E)** FMDV titration at 6 hours post-infection following the treatment with G3Ib or G3Ib’. Data represents mean ± SEM of three independent replicates.

To understand the importance of G3BP1-foci assembly for FMDV replication, we treated infected with FMDV at MOI of 1 for 3.5 hours cells in the presence of G3Ib (or G3Ib’ as control) prior to monitoring G3BP1-foci assembly by immunofluorescence. G3Ib treatment resulted in reduced percentage of cells displaying G3BP1-foci from 47.33 ± 4.81% to 2.57 ± 0.88%, average number per cell from 6.82 ± 2.17 to 0.87 ± 0.57, and average size from 0.19 ± 0.02 to 0.09 ± 0.01 μm^2^, while G3Ib’ had no significant effect (Fig 3A-B). Furthermore, PABP, Caprin1, G3BP2 also no longer accumulate in foci during infection following treatment with G3Ib (S3C Fig), confirming the absence of foci. We assessed the impact of blocking SG formation on FMDV-induced ISR activation by measuring the phosphorylation of eIF2α, puromycin levels, and the cleavage of eIF4GI and G3BP1 (Fig 3C). G3Ib treatment had no impact on FMDV-induced cleavage of host factors or ISR activation when compared to non-treated or G3Ib’-treated samples (Fig 3C). To understand the impact of SGs assembly on FMDV replication and propagation, PK-15 cells were infected with FMDV at MOI of 0.01 and monitored for signs of CPE, reflected by decrease in cell confluency [37] (Fig 3D). Impairing G3BP1-foci assembly did not alter viral replication as the rates of CPE were similar in cells treated with G3Ib, G3Ib’ or untreated cells. The impact on virus replication was investigated by titrating the yield of infection at 6 hpi using plaque assays, revealing similar viral titres and demonstrating no effect of the drugs during infection (Fig 3E). Overall, this supports that FMDV-induced G3BP1-foci do not impact viral replication during infection.

### FMDV-induced G3BP1-foci are not required for IIR activation during infection

**Fig 4:**
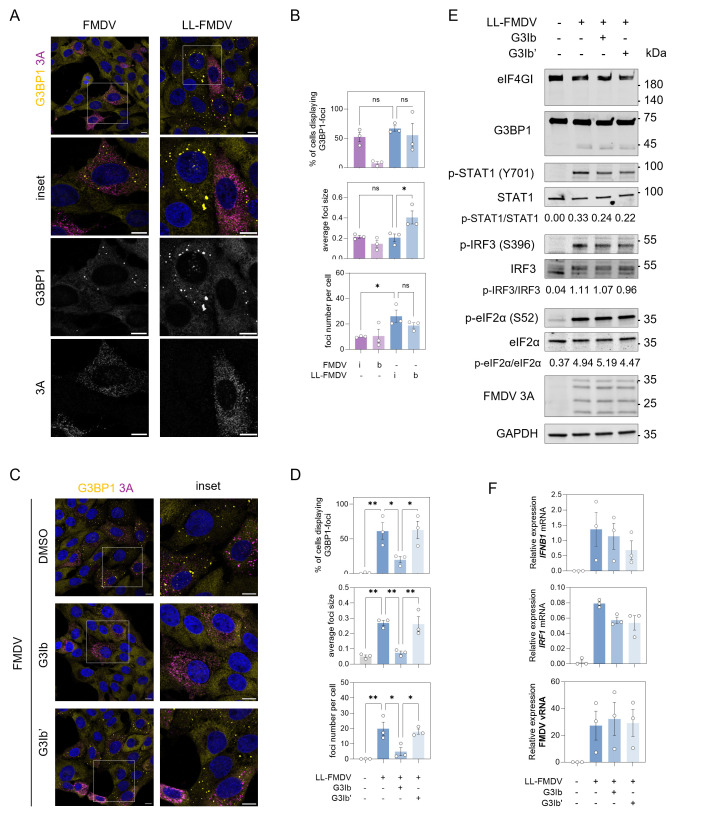
Preventing G3BP1 condensation does not interfere with the activation of interferon response during FMDV infection. **(A-B)** PK-15 cells were infected with FMDV or LL-FMDV for 3.5 hours and analysed by immunofluorescence for the SG markers G3BP1 (gold) and FMDV 3A (magenta). Nuclei were stained with DAPI. Scale bars 10 μm. **(B)** The percentage of cells displaying G3BP1-foci in infected (i) or bystander (b) cells was quantified by manual counting of at least 100 cells per replicate. The average foci size and the number of foci per cell were quantified using the G3BP1 channel and ‘Analyse Particles’ plugin from ImageJ. Results shown as mean ± SEM of at least three independent biological replicates, representing the average of at least 30 cells per condition. *p<0.05; **p<0.01; ns: non-significant; using ordinary one-way ANOVA with Šidák’s multiple comparison post-test. **(C-D)** PK-15 cells were infected with LL-FMDV for 3.5 hours following treatment with G3BP1 condensation inhibitor (G3Ib) or its enactive enantiomer (G3Ib’). The analysis was done as in **(B)**. **(E)** Representative western blot from at least three independent experiments for innate immune response (STAT1 and IRF3 phosphorylation), and integrated stress response (phosphorylation of eIF2α) activation following 8 hours of FMDV infection in the presence of G3Ib or G3Ib’. Molecular weights are indicated on the right. **(F)** Expression levels of *IFNB1*, *IRF1* and *FMDV* genome, were analysed by RT-qPCR with results shown as mean ± SEM, n = 3, normalised to *GAPDH* mRNA.

Although the assembly of G3BP1-foci does not appear to regulate FMDV replication, this does not exclude a role on IIR modulation during infection. The FMDV leader protease (L^pro^) has been previously suggested to counteract SG formation [25] and to contribute to IFN signalling evasion [38–42]. To investigate the interplay between ISR and IIR during FMDV infection, we infected PK-15 cells with an engineered FMDV virus lacking the L^pro^-encoded region (leaderless FMDV, LL-FMDV) [43]. PK-15 cells were infected with WT or LL-FMDV at a MOI of 1 at 3.5 hpi and G3BP1-foci monitored by confocal microscopy. Detailed analysis of foci assembly revealed that LL-FMDV infection resulted in the formation of G3BP1-foci in 66.70 ± 4.62% of infected and 55.30 ± 20.08% of adjacent or bystander cells, whereas the WT virus infection triggered the assembly of G3BP1-foci in 52.30 ± 7.88% of infected but only 7.70 ± 2.60% of bystander cells (Fig 4A-B). Analysis of their number and average size revealed that the G3BP1-foci detected in infected cells with WT and LL-FMDV are similar in size (0.21 ± 0.01 μm^2^ and 0.21 ± 0.04 μm^2^) but more abundant in cells infected with LL-FMDV (9.93 ± 0.37 and 24.4 ± 3.8) (Fig 4B). Upon LL-FMDV infection, these foci appear as abundant (21.37 ± 3.02) but bigger (0.40 ± 0.06 μm^2^) in bystander cells (b) than in infected cells (i) (Fig 4B). Infection with LL-FMDV thus induces the assembly of G3BP1-foci with distinct properties in infected and bystander cells.

To characterise the dynamics and function of LL-FMDV-induced G3BP1-foci in the activation of IIR, we next treated infected cells with G3Ib or G3Ib’ and analysed both G3BP1-foci formation and IIR activation. First, LL-FMDV-induced G3BP1-foci assembly (61.33 ± 12.14%, 19.88 ± 4.37, 0.27 ± 0.02 μm^2^) was impaired by G3Ib treatment (20.00 ± 4.51%, 4.91 ± 2.67, 0.07 ± 0.01 μm^2^), but not G3Ib’ (63.00 ± 12.50%, 17.98 ± 1.68, 0.26 ± 0.05 μm^2^), in both infected and bystander cells (Fig 4C-D). Preventing their assembly did not affect either IIR or ISR signalling after 8 hours of infection, with no observed changes in the phosphorylation levels of IRF3, STAT1, or eIF2α (Fig 4E), or mRNA levels of *IFNB1*, *IRF1*, or FMDV genome (Fig 4F). In contrast, genetic knock-out of G3BP1 resulted in increased FMDV replication (S4 Fig), which suggests a role for G3BP1 during infection independently from their condensation properties.

Overall, this suggests that the assembly of G3BP1-foci during infection is not important for IIR activation or the IFN-mediated response

### FMDV-induced G3BP1-foci are *bona fide* SGs

**Fig 5:**
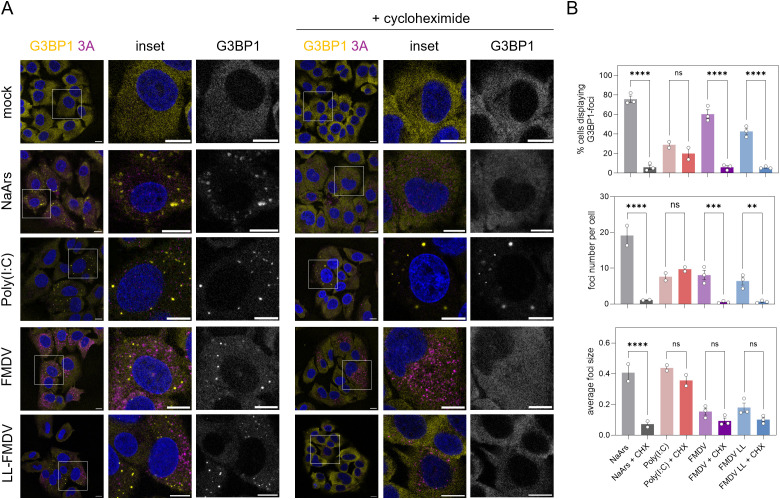
FMDV-induced biocondensates formation are dependent on translation inhibition. PK-15 cells were treated with 1 mM sodium arsenite for 1 h, transfected with 500 ng poly(I:C), or infected with FMDV or LL-FMDV for 3.5 hours, following by a 30-minute treatment with 25 µg/ml cycloheximide treatment prior to harvesting. **(A)** Cells were analysed by immunofluorescence for the SG markers G3BP1 (gold) and viral protein 3A (magenta). Nuclei were stained with DAPI. Scale bars represent 10 μm. **(B)** The percentage of cells displaying G3BP1-foci was quantified by manual counting of at least 100 cells per replicate. The average size of foci per cell and the number of foci per cell were quantified using the G3BP1 channel and ‘Analyse Particles’ plugin from ImageJ. Results shown as mean ± SEM of at least three independent biological replicates, representing the average of at least 30 cells per condition. **p < 0.01; ***p < 0.001; ****p < 0.0001; ns: non-significant; using ordinary one-way ANOVA with Šidák’s multiple comparison post-test.

Compositionally heterogenous SGs have been proposed to assemble in response to different stress triggers, with diverse SG-like condensates assembling during viral infection [44]. To characterize what drives the formation of FMDV-induced foci, we treated infected cells with cycloheximide, an inhibitor of translation elongation that traps mRNA-protein complexes (mRNPs) into polysomes and, therefore blocking protein synthesis and, among other processes prevents the formation of canonical SGs [45]. Treatment with cycloheximide promoted the disassembly of foci induced by sodium arsenite (from 75.67 ± 3.18% to 6.00 ± 2.08% in non-treated and treated samples, respectively), FMDV or LL-FMDV infected cells (from 60.33 ± 4.49% to 6.00 ± 1.53% and from 42.67 ± 3.18% to 5.66 ± 0.66%, respectively), but not by poly(I:C) stimulation (Fig 5A-B). This is supported by a reduction in the number of G3BP1-containing foci following cycloheximide treatment in cells stimulated with sodium arsenite (from 19.17 ± 2.76 to 1.12 ± 0.09) in non-treated and treated samples, respectively) and infected with FMDV (from 8.09 ± 0.59 to 0.53 ± 0.17) or LL-FMDV (from 6.40 ± 1.09 to 0.64 ± 1.32), but not by poly(I:C) stimulation (7.65 ± 1.03 and 8.09 ± 0.59). This suggests that G3BP1-foci formed during FMDV and LL-FMDV infection assemble as a consequence of impaired translation, a property of canonical SGs.

### Preventing SGs disassembly impairs FMDV replication

Given FMDV infection resulted in early assembly of SGs that later disassemble, we hypothesized SGs disassembly may be required for efficient FMDV replication, and thus that SG persistence might impair replication. To test this, PK-15 cells were treated with increasing amounts of raloxifene, previously shown to delay SG dissolution [46]. In PK-15 cells, concentrations equal or higher to 12.5 μM impaired protein translation and lead to the formation of SG (S5A-B Fig), therefore experiments were performed with doses up to 6.25 μM.

**Fig 6:**
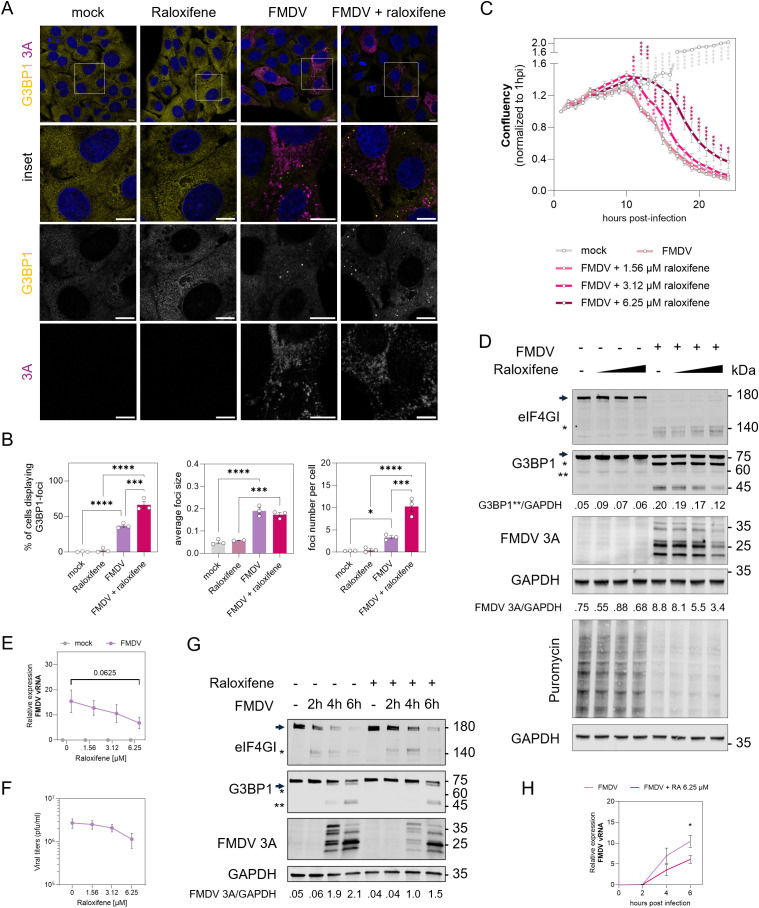
Preventing SGs disassembly impairs FMDV replication. **(A-C)** PK-15 cells were infected with FMDV for 3.5 hours with or without 6.25 µM raloxifene. **(A)** Cells were analysed by immunofluorescence for the SG markers G3BP1 (gold) and viral protein 3A (magenta). Nuclei were stained with DAPI. Scale bars represent 10 μm. **(B)** The percentage of cells displaying SGs was quantified by manual counting of cells with G3BP1-foci of at least 100 cells per replicate. The average size of foci per cell and the number of foci per cell were quantified using the G3BP1 channel and ‘Analyse Particles’ plugin from ImageJ. Results shown as mean ± SEM of at least three independent biological replicates, representing the average of at least 30 cells per condition. *p < 0.05; ***p < 0.001; ****p < 0.0001; using ordinary one-way ANOVA with Šidák’s multiple comparison post-test. **(C)** PK-15 cells were infected with FMDV at a MOI of 0.01 with increasing doses of raloxifene, alongside a mock-infected control, and cell confluence was monitored every hour for 24 hours using an IncuCyte Zoom. Data represents an average of three replicates, normalized to the first time-point measured (1 hpi). **(D)** Representative western blot from at least three independent experiments for eIF4GI and G3BP1 cleavage, viral infection (FMDV 3A), and integrated stress response activation (phosphorylation of eIF2α). Molecular weights are indicated on the right. **(E)** Expression levels of FMDV genome were analysed by qPCR with results shown as mean ± SEM, n = 3, normalised to GAPDH mRNA. **(F)** FMDV titration at 6 hours post-infection following the treatment with increasing concentrations of raloxifene. Data represents the average of three independent replicates. **(G)** Representative western blot of eIF4GI and G3BP1 cleavage and viral infection (FMDV 3A) during a time course of infection in the presence or absence of 6.25 µM raloxifene. Molecular weights are indicated on the right. **(H)** Expression levels of FMDV genome were analysed by qPCR with results shown as mean ± SEM, n = 3, normalised to GAPDH mRNA. *p < 0.05 using two-way ANOVA with Šidák’s multiple comparison between mock-infected or FMDV-infected cells treated with raloxifene and FMDV-infected cells.

PK-15 were infected with FMDV in the presence of raloxifene and SG assembly monitored by immunofluorescence as before (Fig 6A-B). Raloxifene treatment increased both the percentage of infected cells displaying SGs from 37.00 ± 2.08% to 66.33 ± 4.84% (Fig 6B) and the number of SG per cells, from 3.35 ± 0.35 to 10.26 ± 1.19, while their average size did not change (0.19 ± 0.01 and 0.17 ± 0.01 μm^2^) (Fig 6B). Virus-induced CPE was monitored as a measure of replication after infecting PK-15 cells with FMDV at a MOI of 0.01 in the presence of increasing dosed of raloxifene (Fig 6C). Full CPE effect was observed for FMDV-infected cells, untreated or treated with the lowest concentration of raloxifene tested (1.56 μM) at around 14 hpi, whereas treating cells with 3.12 or 6.25 μM at around 16 and 18 hpi, respectively, resulted in reduced CPE indicating attenuated viral replication. At these concentrations, raloxifene did not alter protein synthesis during infection (S5A Fig).

Next, we analysed the effect of raloxifene on FMDV replication, as well as virus-induced cleavage of eIF4GI and G3BP1. PK-15 cells were infected with FMDV at a MOI of 1 for 3.5 hours and either left untreated or treated with increasing amounts of raloxifene at the time of infection. Raloxifene treatment at the highest concentration reduced viral protein expression (Fig 6D), suggesting impaired viral replication. The cleavage of eIF4GI, previously described as L^pro^-driven [35,36], is unaltered by raloxifene, suggesting no effect on L^pro^ activity, while in contrast, the amount of lower molecular weight G3BP1 cleavage product, and not the highest, is reduced (Fig 6D).

A reduction in viral RNA genome was observed during infection following raloxifene treatment at low and high MOI (Figs 6E and S5C), associated with small decrease in infectious virus particles (Figs 6F and S5D). As an alternative approach to observe the drug’s effect, we infected PK-15 cells up to 6 hpi with FMDV at MOI of 1 in the presence of raloxifene at 6.25 µM, which allowed us to confirm that raloxifene delays FMDV infection (Fig 6G-H). As before, the cleavage of eIF4GI during infection remains unaltered with raloxifene treatment, whereas the cleavage of G3BP1, detected here at 4 hpi following FMDV infection, is delayed to 6 hpi by the presence of raloxifene (Fig 6G). This effect can be explained due to the reduced viral translation upon treatment, reflected by a decreased amount of viral genome and protein expression (Fig 6G-H).

Overall, this suggests that inducing persistent condensates with raloxifene slows down FMDV replication kinetics, without having an impact on ISR signalling or host shut-off during infection, which further suggests that the disassembly of G3BP1-foci is contributing to FMDV infection.

### G3BP1 resistant to 3C^pro^ cleavage forms persistent SG during infection

**Fig 7:**
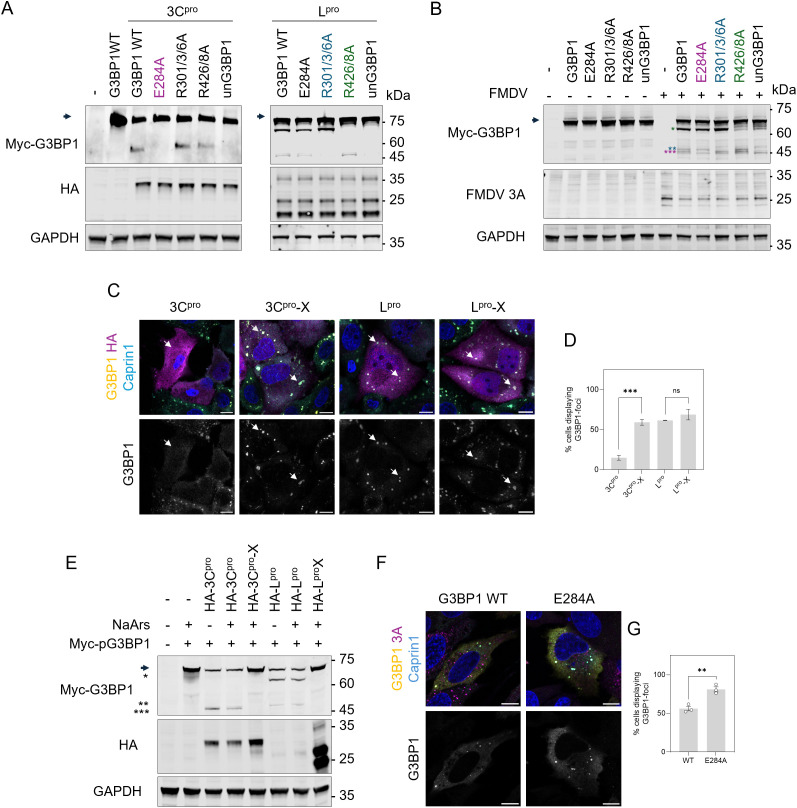
G3BP1 resistant to 3C^pro^ cleavage forms persistent SG during infection. **(A-B)** PK-15 cells were co-transfected with 500 ng of G3BP1 WT or mutant constructs and 500 ng 3C^pro^ or L^pro^ for 24 h or (B) transfected with G3BP1 constructs for 24 h and infected with FMDV for an additional 6 h. **(A-B)** Representative western blot for G3BP1 cleavage following (A) transfection with FMDV proteases or (B) infection. Molecular weights are indicated on the right. **(C)** PK-15 cells transfected with 3C^pro^ or L^pro^, or it’s catalytically inactive forms (3C^pro^-X or L^pro^-X), and treated with sodium arsenite (1 mM for 1 h) were imaged for the SG markers G3BP1 (gold) and Caprin1 (cyan), and HA-tagged viral proteins (magenta). White arrows indicate transfected cells. Nuclei were stained with DAPI. Scale bars represent 10 μm. **(D)** The percentage of cells displaying G3BP1-foci was quantified by manual counting of at least 30 transfected cells per replicate. ***p < 0.001; ns: non-significant; using ordinary one-way ANOVA with Šidák’s multiple comparison post-test. **(E)** Immunoblotting showing G3BP1 cleavage the same conditions as in **(C)**. **(F)** Cells were transfected with 500 nm of G3BP1 WT or E284A following by infection with FMDV were analysed by immunofluorescence for the SG markers G3BP1 (gold) and Caprin1 (cyan), and viral protein 3A (magenta). Nuclei were stained with DAPI. Scale bars represent 10 μm. **(G)** Analysis was performed as in **(D)**. **p < 0.01; using ordinary unpaired t-test.

To better understand the mechanisms leading to virus-induced SG disassembly and their role during FMDV infection, we set out to engineer a G3BP1 protein resistant to cleavage by FMDV proteases. In this work, we show that three distinct G3BP1 cleavage products are detected during FMDV infection (Figs 2C and S6A), which is in agreement with previous reports showing that 3C^pro^ and L^pro^ cleaves G3BP1 in one, and two cleavage sites, respectively [25]. 3C^pro^ was further established to process G3BP1 at the glutamic acid (E) residue at the position 284 (E284) [26]. Based on the putative molecular weight of additional cleavage products and consensus target sequences for L^pro^ [25,26,47] we predicted and introduced point mutations to uncovered the specific L^pro^ cleavage sites in G3BP1 (S6B Fig). Arginine (R) to alanine (A) substitutions at positions 301/303/306 and 426/428 all resulted in impaired L^pro^ proteolytic processing of G3BP1 (S6C Fig). We then engineered a G3BP1 construct bearing all mutations to prevent cleavage by both proteases (unG3BP1). Co-transfection of 3C^pro^ or L^pro^ together with each mutated porcine G3BP1 constructs confirmed that each cleavage site is specific for each protease, whereas the unc-pG3BP1 is no longer cleaved by any (Fig 7A). This supports that 3C^pro^ cleaves G3BP1 at residue E284, as previously described, and highlights residues R301/303/306 and R426/428 as crucial for G3BP1 cleavage by L^pro^.

Additionally, FMDV infection were performed following transfection with G3BP1 constructs for 24 hours (Fig 7B-D). Immunoblotting analysis confirmed the disappearance of the corresponding cleavage product for each mutation (Fig 7B). Of note, the additional product detected close to 45 kDa in all infected conditions following G3BP1 transfection was not observed when co-expressing the viral proteins (Fig 7A), or in endogenous G3BP1 during infection (S6 Fig), and could be attributed to cleavage by cellular proteases associated with cell death as previously suggested [48].

To assert the importance of the distinct protease mediated cleavage, we transfected cells with either 3C^pro^ or L^pro^, or catalytically inactive 3C^pro^ (3C^pro^-X) or L^pro^ (L^pro^-X) mutants, followed by stimulation of SG assembly by sodium arsenite treatment (Fig 7C-E). Cells transfected with 3C^pro^-X or L^pro^-X assembled SGs in 59.4 ± 3.71% and 69.0 ± 6.59% of the transfected cells, respectively. In cells transfected with the corresponding active forms of 3C^pro^ or L^pro^, SG assembled in 15.0 ± 3.09% and 61.7 ± 0.23% respectively, suggesting that 3C^pro^ cleavage is more potent in mediating SG disassembly than L^pro^.

Finally, to confirm the importance of 3C^pro^ cleavage in controlling SG assembly during infection, cells were infected with FMDV following transfected with either wild-type or E284A G3BP1 (Fig 7F-G). Immunofluorescence analysis revealed that SG are present in 56.28 ± 2.35% and 81.23 ± 2.71% of FMDV-infected cells expressing WT or E284A G3BP1.

This suggests that 3C^pro^-mediated cleavage regulates SG disassembly following infection, while L^pro^-mediated cleavage could be involved in preventing additional G3BP1 functions related to its antiviral roles.

## Discussion

Biocondensates formation has emerged as a key process triggered in response to various physiological and pathological challenges to help cells rapidly adjust fundamental processes [49]. Distinct biocondensates with variable functions, including SGs, paracrine granules, RLBs, dsRNA-induced foci (dRIFs), or viral aggregated RNA condensates (VARCs), have been shown to assemble in response to viral infections, with conflicting literature on their roles [44]. Understanding the composition and dynamics of biocondensates is essential to uncover their biological function and relevance for antiviral responses or viral replication.

SGs are among the most studied condensates and have been suggested to act as platforms for innate immune activation in response to viral infection, providing a scaffold for nucleic acids recognition or activation of antiviral signalling [16,22,23,50]. However, recent studies challenged this by demonstrating that SGs do not concentrate antiviral proteins, nor regulate the activation of cell intrinsic antiviral signalling pathways [24,51].

Here, we used distinct viral RNA mimics and FMDV to establish that SGs assembly is uncoupled from the activation of IIR signalling in porcine cells. Our work demonstrates that specifically triggering RIG-I/MAVS-dependent interferon signalling with 3p-hpRNA is not sufficient to promote SG formation (**S1** Fig). We also show that the formation of condensates following poly(I:C) stimulation occurs concomitantly to the activation of IRF3-mediated antiviral signalling and that it further regulates the synthesis of ISGs, such as IRF1 (Figs 1 and **S2**). Cells that assemble condensates show reduced levels of IRF1, likely due to a halted translation state despite active mRNA transcription. A previous report has suggested that *IFNB1* mRNA is stalled within SGs of translationally arrested cells [52], which suggest other ISG mRNAs might also undergo a similar process.

To clarify the importance of these condensates for IIR activation, we show for the first time that chemically impairing the assembly of G3BP1-foci with the condensation inhibitor G3Ib [32] does not impact IIR activation (Fig 1). By preventing G3BP1 self-assembly, G3Ib disrupts the further recruitment and accumulation of translation initiation factors or other SG proteins rates [32] (S2 Fig) and ultimately the assembly of G3BP1-foci, without affecting its RNA-binging activity or the cellular translation initiation rates [32]. This indicates that, in our model, physically preventing the condensation of foci containing G3BP1 and other SG proteins does not impact IIR signalling.

To understand the interplay between G3BP1-foci and IIR activation during viral infection, we focussed our studies on FMDV, a virus previously shown to exploit SGs assembly and evade RLR-mediated IIR [25,38–40]. We characterized the dynamics of biocondensates assembly and disassembly in porcine epithelial cells during infection (Fig 2). Live-cell imaging revealed that G3BP1-foci form early during infection, peaking roughly at 3 hpi, before disassembling prior to initiation of CPE at around 6 hpi. This assembly peak corresponds to the initial timing of eIF2α phosphorylation, which is followed by a later cleavage of G3BP1, suggesting that this might be the trigger for their disassembly (Fig 2).

Our data reconciliate previous reports showing that FMDV modulates SGs formation via G3BP1 cleavage [25,26] and further establish that FMDV promotes SGs disassembly rather than preventing their formation by monitoring their kinetics during infection. These observations are supported by the morphological characterization of the FMDV-induced SGs, which are smaller than the canonical SGs induced by sodium arsenite and resemble the round small foci induced by poly(I:C) (Fig 2). Yet poly(I:C) and FMDV-induced SGs have distinct features and potentially biological roles, given the differences in sensitivity to cycloheximide, suggesting that the latter share some attributes with canonical SGs (Fig 5).

To study the relevance of FMDV-induced SGs during infection on the IIR activation, we took advantage of a FMDV mutant lacking the leader protein (LL-FMDV), which has been shown to downregulate the RIG-I/MDA5 pathway and interfere with the activation of downstream antiviral effectors to impair the IFN-mediated immune response [38,39,42]. FMDV L^pro^ has also been suggested to target G3BP1/2 to suppress SGs [25]. Infection with LL-FMDV allowed us to probe the importance of biocondensates, while the IIR is active (Fig 4). We observed the formation of granules displaying distinct morphology in infected or bystander cells, which paves the way for thorough compositional characterization, to unravel putative differences in biogenesis and/or biological functions. Interestingly, we previously proposed the transfer of stress signalling to bystander cells as a cellular mechanism to limit viral propagation [13]. Therefore, one can speculate that L^pro^ might be involved in suppressing a paracrine mechanism that triggers an antiviral state in bystander cells, leading to the formation of biocondensates. We further show that chemically preventing G3BP1 condensation during infection does not modulate FMDV replication, neither modulates the IIR in LL-FMDV-infected cells, supporting our previous observations that SGs formation and IIR are uncoupled (Fig 4).

Our data further indicates that FMDV remains able to cleave G3BP1 when SG assembly is prevented (Figs 3 and 4), which together with increased replication in G3BP1/2 KO cells (S4 Fig) suggests that FMDV may have evolved to counteract G3BP1 functions that are independent from SG formation. These are all compelling arguments supporting that G3BP1 exert antiviral functions, independently from its condensation properties. This is further supported by previous reports of G3BP1 involvement in promoting the IIR to viral DNA or RNA, and that the absence of G3BP1 in mammalian cells alters IIR activation levels following a dsRNA stimulus [23,53,54]. Furthermore, G3BP1 was shown to interact with FMDV IRES to negatively regulate viral translation, which suggests a further antiviral role for G3BP1 in dampening FMDV replication [55].

To explore whether preventing G3BP1-foci disassembly would interfere with viral infection, we treated the infected cells with raloxifene, previously shown to delay the disassembly of hypoxia-induced SGs [46]. Our data show that raloxifene can delay the disassembly of FMDV-induced G3BP1-foci resulting in delayed infection (Fig 6), making this process a potential target to block FMDV replication. Raloxifene was previously shown to exert an antiviral effect against SARS-CoV-2 [56], influenza A virus [57], hepatitis C virus [58]; however, the underpinning cellular mechanism is yet poorly understood. With previous therapeutic approaches identifying drugs hardening RSV-induced condensates to impair replication, this opens the door for pharmacological targeting of SGs during FMDV infection [59].

The differential effect of G3BP1 cleavage products during infection following raloxifene treatment (Fig 6) suggest distinct roles for both proteases in G3BP1 proteolytic targeting and SG disassembly. In fact, L^pro^ activity does not seem to be affected by raloxifene as eIF4GI cleavage remains unaltered. Although we and others [25] have shown that G3BP1 is cleaved by FMDV both L^pro^ and 3C^pro^ during infection, our results further establish that 3C^pro^, but not L^pro^, is responsible for promoting SG disassembly (Figs 6 and **7**).

Other members of the *Picornaviridae* family have shown to modulate SG dynamics and cleave G3BP1. Seneca Valley virus (SVV), which also causes vesicular disease in pigs, transiently induces SG formation at earlier stages of infection, in a PKR-eIF2α-dependent manner, and inhibits SGs at later stages through 3C^pro^-mediated disruption of eIF4GI-G3BP1 interaction [60]. Similar dynamics are observed in poliovirus [61], coxsackievirus B3 (CVB3) [62], and enterovirus 71 (EV71) [63,64], which all induce SGs at an early stage but limit SGs as infection progresses, a trait associated with 3C^pro^-mediated G3BP1 cleavage. Poliovirus, EV71, and CVB3 3C^pro^ cleave G3BP1 at the residue Q325/Q326 [61–63,65], while FMDV 3C^pro^ cleaves it at the residue E284. Both residues belong to the proline-rich motif (PxxP) intrinsically disordered region 2 (IDR2) of G3BP1, a domain that fine-tunes its function, including SG assembly, dynamics and composition [66]. Other picornavirus proteases, including poliovirus [61], CBV3 [62], and enterovirus [63] 2A^pro^, or the EMCV leader (with no protease activity) [65] have shown to have no effect on G3BP1 cleavage or SG disassembly.

In this study, we mapped the cleavage sites for L^pro^ to be at residues R301/3/6 and R426/8 (Figs 7 and S5) that belong, respectively, to the PxxP IDR2 and arginine-glycine rich box (RGG) IRD3 domains. Alterations in the IDR2 was shown to modulate G3BP1 RNA-binding capacity, whereas removal of IDR3 was shown to abolish RNA binding and impaired ability to support SG assembly [66]. Furthermore, G3BP1 was shown to interact with RIG-I to enhance interferon-mediated responses via its RGG domain [53], which lead us to speculate that the initial cleavage induced by L^pro^ at the R426/8 residues might prevent this interaction to dampen antiviral responses.

Altogether, this suggests that during FMDV infection, both L^pro^ and 3C^pro^ act together to prevent antiviral activities exerted by G3BP1 and by promoting the disassembly of SG that could become antiviral platforms at later stages of infection.

In conclusion, we established for the first time in a porcine cell model that the antiviral response to dsRNA or an RNA virus infection and the assembly of biocondensates – including canonical FMDV-induced SGs or cycloheximide-resistant poly(I:C)-driven condensates – are triggered independently, and that FMDV has evolved differential cleavage of G3BP1 by L^pro^ and 3C^pro^ to regulate its functions.

## Materials and methods

### Cell culture

PK-15 cell lines were grown in Dulbecco’s modified Eagle’s medium (DMEM, Sigma-Aldrich, #D5796) supplemented with 10% foetal bovine serum (FBS) (Life Science Production), 100 U/ml penicillin and 100 μg/ml streptomycin (Sigma Aldrich), and 1 mM sodium pyruvate (Gibco), in a humidified incubator at 37ºC and 5% CO_2_ environment. BHK-21 cells were grown in Glasgow’s MEM (GMEM, Sigma-Aldrich, #G5154) supplemented with 10% FBS, 100 U/ml penicillin and 100 μg/ml streptomycin, 2mM L-glutamine (Sigma-Aldrich), and 5% Tryptose Phosphate Broth (TPB, Sigma Aldrich, #18050-039).

PK-15 G3BP1-RFP cells were generated following the guidelines of the TrueTag Donor DNA Kit, RFP using a custom gRNA (5’-CGAAGAGCAATTCACTGCCT-3’) and homology arm primers, forward 5’- CAGGATTTGGAGTGGGAAGGGGGCTTGCGCCACGGCAGGGAAGTGGCTCAGGTTCTGGA-3’ and reverse 5’- GACAGGGTTTGTATTGTTGCGCGAAGAGCAATTCACTTGGCCGATCGCATACAGAG-3’, targeting the C-terminal of porcine G3BP1 (*Sus scrofa*, NM_001205405.1). Cells were grown with complete DMEM supplemented with 5 μg/ml of blasticidin (InvivoGen) to ensure selection. Single cell clones were expanded and selected for RFP and G3BP1 assembly following stress treatments. To proceed with the live-cell experiments, four clones were pooled together in equal proportions.

PK-15 G3BP1/2 KO cells were generated using a three-gRNA strategy to delete a portion of each locus encoding the corresponding protein. gRNAs (Table 1) (Synthego) were resuspended in 1x TE buffer (10 mM Tris-HCl 0.1 mM EDTA, pH 7.5) to a final concentration of 100 μM (100 pmol/μl). The assembly of gRNA/Cas9 ribonucleoprotein complexes (RNPs) were obtained by gently mixing gRNAs with recombinant Alt-R Cas9 protein (Integrated DNA Technologies, IDT) at a 9:1 ratio in Nucleofector solution from the SF Cell Line Nucleofector Kit (Lonza), followed by 10 min incubation at room temperature. PK-15 cells (1E + 06) were resuspended in 25 μl of Nucleofection solution and mixed with 25 μl of each pre-assembled RNPs. Nucleofection was performed using an Amaxa 4D nucleofector (Lonza). Nucleofected cells were incubated for 48 h prior to clonal amplification and screening for homozygous clones using target-specific PCR (GoTaq Hot Start, Promega) on genomic DNA using the correspondent primers (Table 2). After genotyping, expression levels of each protein in controls and KO single-cell clones were assessed by western blotting. After characterization and for further experiments, cell pools were generated by mixing three clones in equal proportions.

**Table 1:** gRNA sequences used for the KO cells.

Gene	gRNA sequences (5’-3’)
G3BP1	GAACUCUUCUUAUGUCCAUG; GCGGAUCUUGGUGUGGCAGU; AGCCACCAAAGACCUCAUCU
G3BP2	CCGCCCUACAAGCAGCGGAC; CAUGGUGGAGUAGAUGCUAG; GCUCAUGCAACCUUGAGUGA

**Table 2:** Primers used for the characterization of KO cells.

Primer	Primer sequences (5’-3’)
ko_pG3BP1_scr_for	CTGGGAGGCAGATAGGTAACG
ko_pG3BP1_scr_rev	CTTCTGCCACTATCCACTAAGC
ko_pG3BP2_scr_for	GAGGAGATGGCAAATGTTGGA
ko_pG3BP2_scr_rev	GACAGCAAACCCATGACCTG

### Chemical treatments

Sodium arsenite (Sigma-Aldrich) was diluted in H_2_O and used at a concentration of 1 mM for 1 h. Low-molecular weight poly(I:C) (Sigma-Aldrich) diluted in H_2_O, 150 mM NaCl, and 3p-hpRNA (InvivoGen) diluted in nuclease-free water, were transfected using lipofectamine 2000 (Thermo Fisher Scientific) at 1:3 in Opti-MEM (Thermo Fisher Scientific), according to manufacturer’s instructions. G3Ib or the inactive form G3Ib’, were reconstituted in DMSO and added to the cells 20 minutes prior to treatments or at the time of the infection, at a concentration of 50 µM. Cycloheximide (Sigma Aldrich) was reconstituted in ethanol and added to the cells at 25 μg/ml, 30 minutes prior to sample harvesting. Raloxifene (Cambridge Bioscience) was reconstituted in DMSO and added to the cells at the time of the infection at 1.5 – 6.25 µM.

### Transfection

Transfection of plasmids listed in Table 3. was performed using lipofectamine 2000 (Thermo Fisher Scientific) at 1:2 in Opti-MEM (Thermo Fisher Scientific), according to manufacturer’s instructions. 500 ng of each plasmid was used to transfect cells in a 24-well for 24 hours. When followed by infection, transfection media was changed to fresh DMEM 6 hours after transfection.

**Table 3:** Plasmids used in the transfection experiments.

Construct	Backbone	Reference
Myc-pG3BP1-FLAG	pTwist cDNA 3.1	This work
Myc-pG3BP1-E284A-FLAG	pTwist cDNA 3.1	This work
Myc-pG3BP1-R295/8A-FLAG	pTwist cDNA 3.1	This work
Myc-pG3BP1-R301/3/6A-FLAG	pTwist cDNA 3.1	This work
Myc-pG3BP1-R313/6/9A-FLAG	pTwist cDNA 3.1	This work
Myc-pG3BP1-R415/8A-FLAG	pTwist cDNA 3.1	This work
Myc-pG3BP1-R426/8A-FLAG	pTwist cDNA 3.1	This work
Myc-pG3BP1-uncl-FLAG	pTwist cDNA 3.1	This work
EMCV IRES-FMDV 3C^pro^	pTwist CMV BG WPRE Neo	This work
EMCV IRES-FMDV 3C^pro^-X	pTwist CMV BG WPRE Neo	This work
HCV IRES-FMDV L^pro^	pTwist CMV BG WPRE Neo	This work
HCV IRES-FMDV L^pro^-X	pTwist CMV BG WPRE Neo	This work

### FMDV infections

FMDV and LL-FMDV viruses were recovered from BHK-21 cell after full CPE in GMEM supplemented with 1% FBS (Thermo Fisher), 100 U/ml penicillin and 100 μg/ml streptomycin, 2 mM L-glutamine, and 5% TBP. Viral titres were then determined by plaque assays. BHK-21 cell monolayers were infected with 10-fold serial dilutions of virus stock, overlaid with Eagle overlay media supplemented with 5% TBP 100 U/ml penicillin and 100 μg/ml streptomycin (Sigma Aldrich), and 0.6% Indubiose (MP Biomedicals), and incubated for 72 hours at 37°C. Cells were fixed and stained with 1% (w/v) methylene blue in 10% (v/v) ethanol and 4% formaldehyde in PBS.

For infection experiments, PK-15 cells were seeded in 24-well plates and inoculated with 150 μl of virus dilution in DMEM supplemented with 1% FBS, 100 U/ml penicillin and 100 μg/ml streptomycin, 2 mM L-glutamine and 1 mM sodium pyruvate. After 30 minutes, the inoculum was removed and 500 μl of fresh media were added to the cells with the correspondent treatment, when mentioned. The development of CPE was monitored every hour using the Incucyte S3 Live-Cell Analysis System (Essen BioScience) and imaged using phase light every four hours until complete CPE was reached (35).

### Immunofluorescence microscopy and live cell experiments

For immunofluorescence assays, PK-15 or PK-15 G3BP1/2 KO cells were seeded on sterilized coverslips and fixated with paraformaldehyde after experiments. Media was removed and fixed with 4% paraformaldehyde (PFA) in PBS for 20 min at RT. Fixation solution was removed, coverslips were rinsed with PB and stored at 4 ºC or processed immediately. Cells were permeabilized with 0.1% Triton X-100 (Sigma-Aldrich) in PBS for 5 min at RT and blocked with 0.5% bovine serum albumin (Sigma-Aldrich) in PBS for 1h at RT. Cells were then incubated with primary antibody (Table 4) for 1h at RT, washed 3 times with PBS and incubated with secondary antibody solution containing 0.2 μg/ml DAPI for 1h at RT. Secondary antibodies used were goat anti-rabbit or goat anti-mouse Alexa 488, 555 or 647 (1:500, Invitrogen). When mentioned, phalloidin 647-conjugated (1:500, SC-363797) was also added. Coverslips were then rinsed 3 times with PBS and mounted onto microscope slides with 5 μl Mowiol 4-88 (Sigma-Aldrich). Cells were visualized and imaged with Leica STELLARIS 5 inverted microscope or Leica SP8 CLSM upright microscope using a 60X objective.

**Table 4:** Antibodies used on immunofluorescence staining.

Primary antibodies used in IFA	Host species	Reference
IRF3	Rabbit	Cell Signalling Technology, #8478
IRF1	Rabbit	Cell Signalling Technology, #8784
Puromycin	Mouse	Millipore, MABE343
G3BP1	Rabbit	Merck, G6046
G3BP1	Mouse	BD Transduction, #611127
G3BP2	Rabbit	Abcam, ab86135
PABP	Rabbit	Abcam, ab21060
Caprin1	Rabbit	ProteinTech, 15112-1-AP
eIF4GI	Rabbit	Cell Signalling Technology, #2858
HA-tag	Mouse	Cell Signalling Technology, # 2276
FMDV 3A	Mouse	Clone 2C2

The percentage of cells displaying SGs was quantified by manual counting of cells with G3BP1-foci of at least 100 cells per replicate. The average size of foci per cell and the number of foci per cell were quantified using the G3BP1 channel and ‘Analyse Particles’ plugin from ImageJ. Results shown as mean ± SEM of at least three independent biological replicates, representing the average of at least 30 cells per condition unless stated otherwise.

For live experiments, PK-15 G3BP1-RFP cells were seeded into Nunc Lab-Tek II chambered cover glasses (Thermo Fisher). The day after, cells were infected and, after inoculation, incubated with 10 μg/ml Hoechst H3570 (Thermo Fisher) for 30 minutes, and imaged for up to 7 hours after infection in Leibovitz’s L-15 Medium (Thermo Fisher) supplemented with 1% FBS. Cells were visualized and movies were made with Leica SP8 CLSM inverted microscope using a 40X objective. Confocal images were analysed utilizing Image J by following the assembly and disassembly of G3BP1-foci in cells that develop CPE at around 6 hours of infection.

### Immunoblotting

Cells were seeded in 24-well plates to be around 80% confluent at the indicated times. Cells were treated as required, then media was removed, washed with cold PBS, lysed with 1x red Loading buffer supplemented with DTT (Cell Signalling) and boiled at 95 ºC for 5 min. Lysates were separated in 4–20% gradient gel with 1x SDS running buffer (25 mM Tris, 192 mM glycine, and 0.1% SDS) and proteins were transferred to a nitrocellulose membrane using the Pierce Power Station and 1-step Transfer Buffer (Thermo Scientific), or by wet transfer using an appropriate buffer (25 mM Tris, 192 mM glycine, and 20% methanol). Membranes were blocked in 5% BSA in TBS-Tween (TBS-T) for 1 h at RT and then incubated with primary antibodies (Table 5) overnight at 4 ºC. Next day, membranes were washed 3 times with TBS-T and subsequently incubated with secondary antibodies (Dako 1:5000, or LiCor IRDye 1:10,000) diluted in blocking buffer for 1 h at RT. After three washes with TBS-T, secondary antibodies were labelled with Clarity Western ECL Substrate (Bio-Rad) when required, and membranes were imaged using Bio-Rad or Odyssey Imaging systems. Band intensity was quantified and analysed using Image Studio Lite (Ver.5.2). Total protein was detected using ponceau S staining.

**Table 5:** Antibodies used on western blot staining.

Primary antibodies used in WB	Host species	Reference
p-STAT1 (Y701)	Rabbit	Cell Signalling Technology, #9167
STAT1	Mouse	Cell Signalling Technology, #9176
p-IRF3 (S396)	Rabbit	Cell Signalling Technology, #4947
IRF3	Rabbit	Cell Signalling Technology, #8478
IFIT2	Rabbit	Protein Tech, 12604-1-AP
IRF1	Rabbit	Cell Signalling Technology, #8784
ISG15	Rabbit	Protein Tech, 15981-1-AP
p-eIF2α (S52)	Rabbit	Cell Signalling Technology, #3597
eIF2α	Rabbit	Cell Signalling Technology, #9722
Puromycin	Mouse	Millipore, MABE343
GAPDH	Mouse	Ambion, AM4300
G3BP1	Rabbit	Merck, G6046
G3BP1	Mouse	BD Transduction, #611127
G3BP2	Rabbit	Abcam, ab86135
eIF4GI	Rabbit	Cell Signalling Technology, #2858
FMDV 3A	Mouse	Clone 2C2
Myc-tag	Mouse	Cell Signalling Technology, #2276
HA-tag	Rabbit	Cell Signalling Technology, C29F4

### Preparation of RNA samples and RT-qPCR

Cells were seeded in 24-well plates to be around 80% confluent at the indicated times. Cells were treated as needed, washed with cold PBS, and total RNA was extracted using a QiaGen RNAeasy kit (QiaGen, #74104) following the manufacturer’s instructions. Purified RNA was quantified on Nanodrop, and 500 ng of RNA was subject to reverse transcription using M-MLV Reverse Transcriptase (Invitrogen, #28025013). Next, real-time quantitative PCR (qPCR) was performed in duplicate with 25 ng of template, specific primers (Table 6) used at a final concentration of 0.25 μM and PowerUp SYBR Green Master Mix (Applied Biosystems), following manufacturer’s instructions, using the Quant Studio 5 software (Applied Biosystems). The comparative Ct method was used; ΔCT values were obtained by normalizing the Ct values from genes of interest to *GAPDH*, and the 2-ΔCT method was used to calculate its relative expression in each sample.

**Table 6:** List of primers used for RT-qPCR analyses.

qPCR primers	Sequence 5’-3’
IFNB-FW_Sus scrofa	GCTAACAAGTGCATCCTCCAAA
IFNB-RV_Sus scrofa	AGCACATCATAGCTCATGGAAAGA
ISG15-FW_Sus scrofa	GATCGGTGTGCCTGCCTTC
ISG15-RV_Sus scrofa	CGTTGCTGCGACCCTTGT
ISG56-FW_Sus scrofa	AAATGAATGAAGCCCTGGAGTATT
ISG56-RV_Sus scrofa	AGGGATCAAGTCCCACAGATTTT
IRF1-FW_Sus scrofa	AGTCCAAGTCCAGCCGAGAT
IRF1-RV_Sus scrofa	TACTGATGGCACACGGTGAC
IL6-FW_Sus scrofa	CTGCTTCTGGTGATGGCTACTG
IL6-RV_Sus scrofa	GGCATCACCTTTGGCATCTT
PKR-FW_Sus scrofa	AAAGCGGACAAGTCGAAAGG
PKR-RV_Sus scrofa	TCCACTTCATTTCCATAGTCTTCTGA
GAPDH-FW_Sus scrofa	ACATGGCCTCCAAGGAGTAAGA
GAPDH-RV_Sus scrofa	GATCGAGTTGGGGCTGTGACT
FMDV genome-FW	ACTGGGTTTTACAAACCTGTGA
FMDV genome-RV	GCGAGTCCTGCCACGGA

### Ribopuromycyclation assay

To capture translation efficacy, cells were treated with 10 μg/ml of puromycin (Sigma-Aldrich) for 5 min at 37 C to label the nascent polypeptide chains before addition of 180 μM of emetine (Sigma-Aldrich) to block the translation elongation with a further incubation of 2 min at room temperature. Puromycin incorporation was quantified by immunoblotting or immunofluorescence as described before (13).

### Statistical analyses

Statistical analyses were performed using GraphPad Prism software (Ver 10) and at least three biological replicates were analysed in the experiments. Statistical tests performed and correspondent significance are indicated in the Fig legends.

## Supporting information

S1 Fig IIR is robustly induced following PKR and RIG-I stimulation in PK-15 cells. PK-15 cells were transfected with increasing amounts of 3p-hpRNA or poly(I:C) for 6 hours. Transfection with lipofectamine was used as a control. (A) Expression levels of *IFNB1* and ISGs were analysed by RT-qPCR with results shown as mean ± SEM, n=3, normalised to *GAPDH* mRNA. *p<0.05, **p<0.1, *** p<0.001, ****p<0.0001; using two-way ANOVA with Šidák’s multiple comparison between 3p-hpRNA (in green) or poly(I:C) (in red) and lipofectamine. (B) Representative western blot and quantification from at least three independent experiments for markers of interferon signalling activation (STAT1 and IRF3 phosphorylation levels), interferon-stimulated genes expression (IFIT2, IRF1 and ISG15), and integrated stress response activation (phosphorylation of eIF2α and puromycin levels). Molecular weights are indicated on the right. Band intensities were normalised to that of total protein or GAPDH. (C) PK-15 cells were stimulated with increasing concentrations of 3p-hpRNA for 6 h. Cells were analysed by immunofluorescence for the SG markers G3BP1 (gold) and eIF4GI (magenta), and F-actin marker phalloidin (cyan). Nuclei were stained with DAPI. Scale bars represent 10 μm. The percentage of cells displaying SGs was quantified by manual counting of at least 100 cells per replicate. Data represents mean ± SEM of three biological replicates.
(TIF)

S2 Fig Characterization of poly(I:C) stimulation of IIR and ISR in PK-15 cells. (A) Confocal images of PK-15 cells transfected with 500 ng poly(I:C) up to 8 hours post stimulation. Cells were analysed by immunofluorescence for the SG marker G3BP1 (gold), innate immune response activation markers IRF3 or IRF1 (magenta), and F-actin marker phalloidin (cyan). Nuclei were stained with DAPI. Scale bars represent 10 μm. (B) Quantification of the percentage of cells displaying G3BP1-foci, number of foci per cell, average number and size of foci per cell. Data represents mean ± SEM, n=3. (C) Quantification of IRF1 or IRF3 nuclear intensities in cells positive or negative for SGs was done using ImageJ. *p<0.05, **p<0.01, *** p<0.001; using two-way ANOVA with Šidák’s multiple comparison between cells with (foci+) and without (foci-) G3BP1 condensates at each time point. (D) PK-15 were stimulated with poly(I:C) for 6 h in the presence of ruxolitinib, an inhibitor if interferon signalling. Cells were analysed by immunofluorescence for the SG marker G3BP1 (gold) and innate immune response activation marker IRF1 (magenta). Nuclei were stained with DAPI. Scale bars represent 10 μm. Quantification of IRF1 nuclear intensities was done using ImageJ considering at least 100 cells per sample. (E) Cells incubated with G3Ib (or G3Ib’) followed by sodium arsenite treatment (1 mM for 1 h) were analysed by immunofluorescence for the SG markers G3BP1 (gold) and as referred (cyan). Nuclei were stained with DAPI. Scale bars represent 10 μm. (F) Expression levels of *IFNB1* and ISGs were analysed by RT-qPCR with results shown as mean±SEM, n=3, normalised to GAPDH mRNA. **p<0.01; ***p<0.001; ****p<0.0001; ns: non-significant; using ordinary one-way ANOVA with Šidák’s multiple comparison post-test.
(TIF)

S3 Fig Comparison of infection-mediated cell signalling between PK-15 and PK-15 G3BP1-RFP. (A) PK-15 G3BP1-RFP cells were treated with sodium arsenite (1 mM for 1 h) or infected with LL-FMDV for 3.5 hours. Cells were analysed by immunofluorescence for the endogenous G3BP1 (gold) and G3BP1-RFP (magenta). Nuclei were stained with DAPI. Scale bars represent 10 μm. (B) Representative western blot for eIF4GI and G3BP1 cleavage, viral infection (FMDV 3A), and integrated stress response activation (phosphorylation of eIF2α and puromycin). Molecular weights are indicated on the right. (C) PK-15 cells were infected with FMDV for 3.5 hours following treatment with G3Ib. Cells were analysed by immunofluorescence for FMDV 3A (magenta), G3BP1 (gold) and a second SG marker, including PABP, caprin1, G3BP2 and eIF4GI (cyan). Nuclei were stained with DAPI. Scale bars represent 10 μm.
(TIF)

S4 Fig FMDV replication is enhanced in the absence of G3BP1/2. (A) PK-15 NTC or G3BP1/2 KO cells were treated with 1 mM sodium arsenite for 1 h or stimulated with 500 ng poly(I:C) for 4 h. Cells were analysed by immunofluorescence for the SG markers G3BP1 (gold) and G3BP2 or the innate immune response activation markers IRF3 (magenta). Nuclei were stained with DAPI. Scale bars represent 10 μm. (B-C) PK-15 NTC or G3BP1/2 KO cells were transfected with 3p-hpRNA or poly(I:C), or infected with FMDV or LL-FMDV up to 6 hours. (B) Representative immunoblotting of innate immune response activation (STAT1 phosphorylation) and viral protein levels (FMDV 3A). Molecular weights are indicated on the right. Band intensities were normalised to that of total protein or GAPDH. (C) Expression levels of *IFNB1* and ISGs were analysed by RT-qPCR with results shown as mean ± SEM, n=3, normalised to GAPDH mRNA. ****p<0.0001; using two-way ANOVA with Šidák’s multiple comparison post-test.
(TIF)

S5 Fig Concentrations of raloxifene higher than 6.25 µM induce translational shut-off and stress granules formation. PK-15 cells were treated with increasing concentrations of raloxifene and infected with FMDV at a low or high MOI for the indicated times. (A) Representative western blot for puromycin, indicating translation levels, G3BP1 cleavage and viral infection (FMDV 3A). Molecular weights are indicated on the right. (B) Cells treated with 6.25 or 12.5 μM of raloxifene were analysed by immunofluorescence for the SG marker G3BP1 (gold). Nuclei were stained with DAPI. Scale bars represent 10 μm. (C-D) PK-15 cells were treated with increasing amounts of raloxifene and infected with FMDV at a MOI of 0.01. (C) Expression levels of FMDV genome were analysed by qPCR with results shown as mean ± SEM, n=3, normalised to GAPDH mRNA. (D) FMDV titration at 6 hours post-infection following the treatment with increasing concentrations of raloxifene. Data represents the average of three independent replicates. **p<0.1, *** p<0.001, using two-way ANOVA with Šidák’s multiple comparison between mock-infected or FMDV-infected cells treated with raloxifene and FMDV-infected cells.
(TIF)

S6 Fig G3BP1 is cleaved by FMDV L^pro^ in the residues R301/303/306 and R426/428. (A) Representative western blot showing G3BP1 cleavage upon FMDV infection. Molecular weights of full length G3BP1 and cleavage products are indicated on the right (*, ** and ***). (B) Available sequence of porcine G3BP1 from NCBI (NP_001192334.1) and representative structure cartoon, highlighting the cleavage site established for 3C^pro^ (in pink) and the predicted sites for L^pro^ (in blue and greed). (C) Representative western blot of G3BP1 cleavage by L^pro^ following co-transfection of viral L^pro^ and mutated G3BP1 constructs. Molecular weights are indicated on the right and cleavage products (* and **) or unspecific band (#) are indicated on the right. When missing, the cleavage products are represented with asterisks (* or **) with the correspondent colour to the mutations (blue or green).
(TIF)
