# Supplementary figures and images for: The assembly of stress granules during foot-and-mouth disease virus infection is uncoupled from activation of cellular intrinsic antiviral signalling
Source: PLoS Pathog. 2026 Jun 11;22(6):e1013722. doi: 10.1371/journal.ppat.1013722 (PMC13274924; doi:10.1371/journal.ppat.1013722)

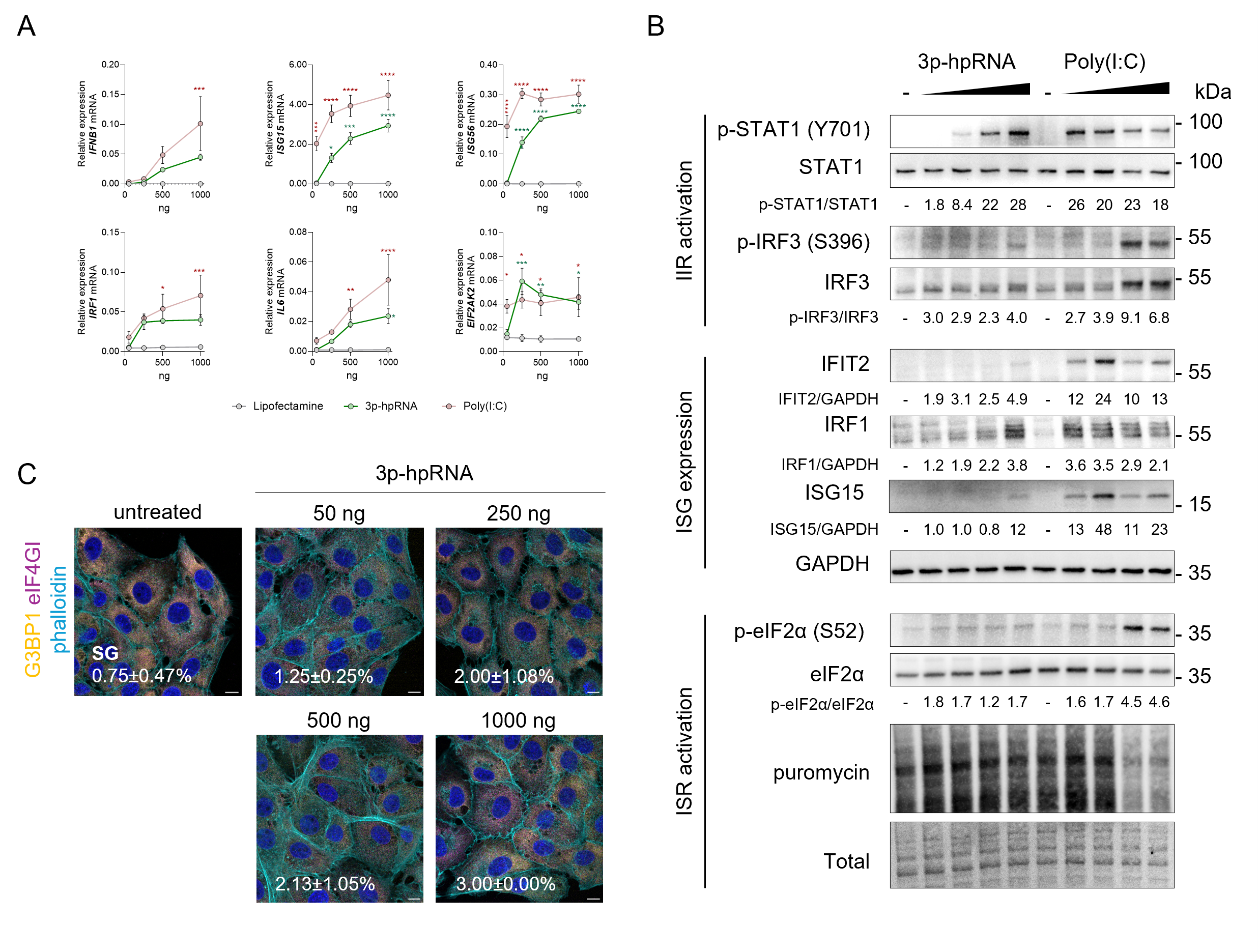

Supplement: S1 Fig — PK-15 cells were transfected with increasing amounts of 3p-hpRNA or poly(I:C) for 6 hours. Transfection with lipofectamine was used as a control. (A) Expression levels of IFNB1 and ISGs were analysed by RT-qPCR with results shown as mean ± SEM, n=3, normalised to GAPDH mRNA. *p<0.05, **p<0.1, *** p<0.001, ****p<0.0001; using two-way ANOVA with Šidák’s multiple comparison between 3p-hpRNA (in green) or poly(I:C) (in red) and lipofectamine. (B) Representative western blot and quantification from at least three independent experiments for markers of interferon signalling activation (STAT1 and IRF3 phosphorylation levels), interferon-stimulated genes expression (IFIT2, IRF1 and ISG15), and integrated stress response activation (phosphorylation of eIF2α and puromycin levels). Molecular weights are indicated on the right. Band intensities were normalised to that of total protein or GAPDH. (C) PK-15 cells were stimulated with increasing concentrations of 3p-hpRNA for 6 h. Cells were analysed by immunofluorescence for the SG markers G3BP1 (gold) and eIF4GI (magenta), and F-actin marker phalloidin (cyan). Nuclei were stained with DAPI. Scale bars represent 10 μm. The percentage of cells displaying SGs was quantified by manual counting of at least 100 cells per replicate. Data represents mean ± SEM of three biological replicates. (TIF) [file ppat.1013722.s001.tif]

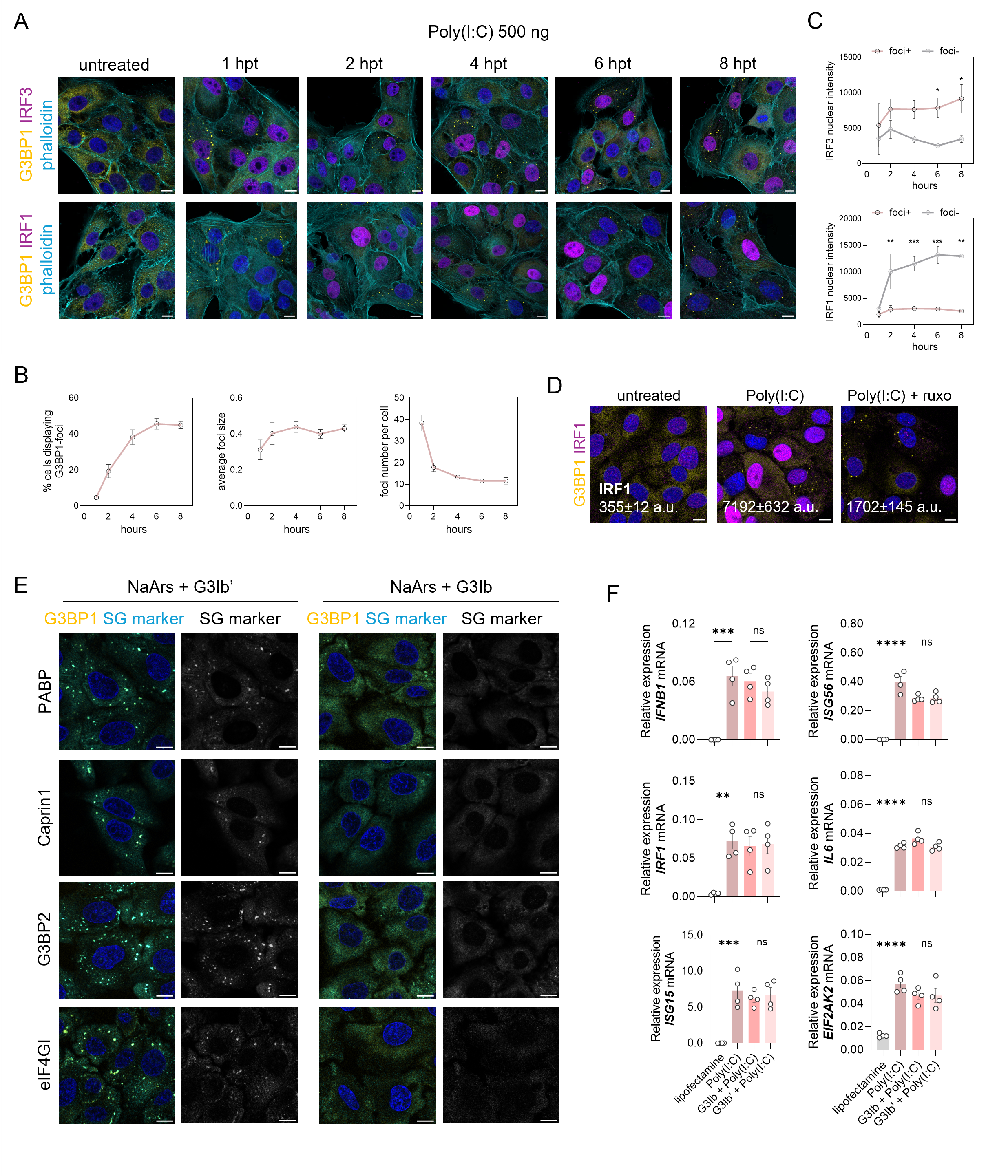

Supplement: S2 Fig — (A) Confocal images of PK-15 cells transfected with 500 ng poly(I:C) up to 8 hours post stimulation. Cells were analysed by immunofluorescence for the SG marker G3BP1 (gold), innate immune response activation markers IRF3 or IRF1 (magenta), and F-actin marker phalloidin (cyan). Nuclei were stained with DAPI. Scale bars represent 10 μm. (B) Quantification of the percentage of cells displaying G3BP1-foci, number of foci per cell, average number and size of foci per cell. Data represents mean ± SEM, n=3. (C) Quantification of IRF1 or IRF3 nuclear intensities in cells positive or negative for SGs was done using ImageJ. *p<0.05, **p<0.01, *** p<0.001; using two-way ANOVA with Šidák’s multiple comparison between cells with (foci+) and without (foci-) G3BP1 condensates at each time point. (D) PK-15 were stimulated with poly(I:C) for 6 h in the presence of ruxolitinib, an inhibitor if interferon signalling. Cells were analysed by immunofluorescence for the SG marker G3BP1 (gold) and innate immune response activation marker IRF1 (magenta). Nuclei were stained with DAPI. Scale bars represent 10 μm. Quantification of IRF1 nuclear intensities was done using ImageJ considering at least 100 cells per sample. (E) Cells incubated with G3Ib (or G3Ib’) followed by sodium arsenite treatment (1 mM for 1 h) were analysed by immunofluorescence for the SG markers G3BP1 (gold) and as referred (cyan). Nuclei were stained with DAPI. Scale bars represent 10 μm. (F) Expression levels of IFNB1 and ISGs were analysed by RT-qPCR with results shown as mean±SEM, n=3, normalised to GAPDH mRNA. **p<0.01; ***p<0.001; ****p<0.0001; ns: non-significant; using ordinary one-way ANOVA with Šidák’s multiple comparison post-test. (TIF) [file ppat.1013722.s002.tif]

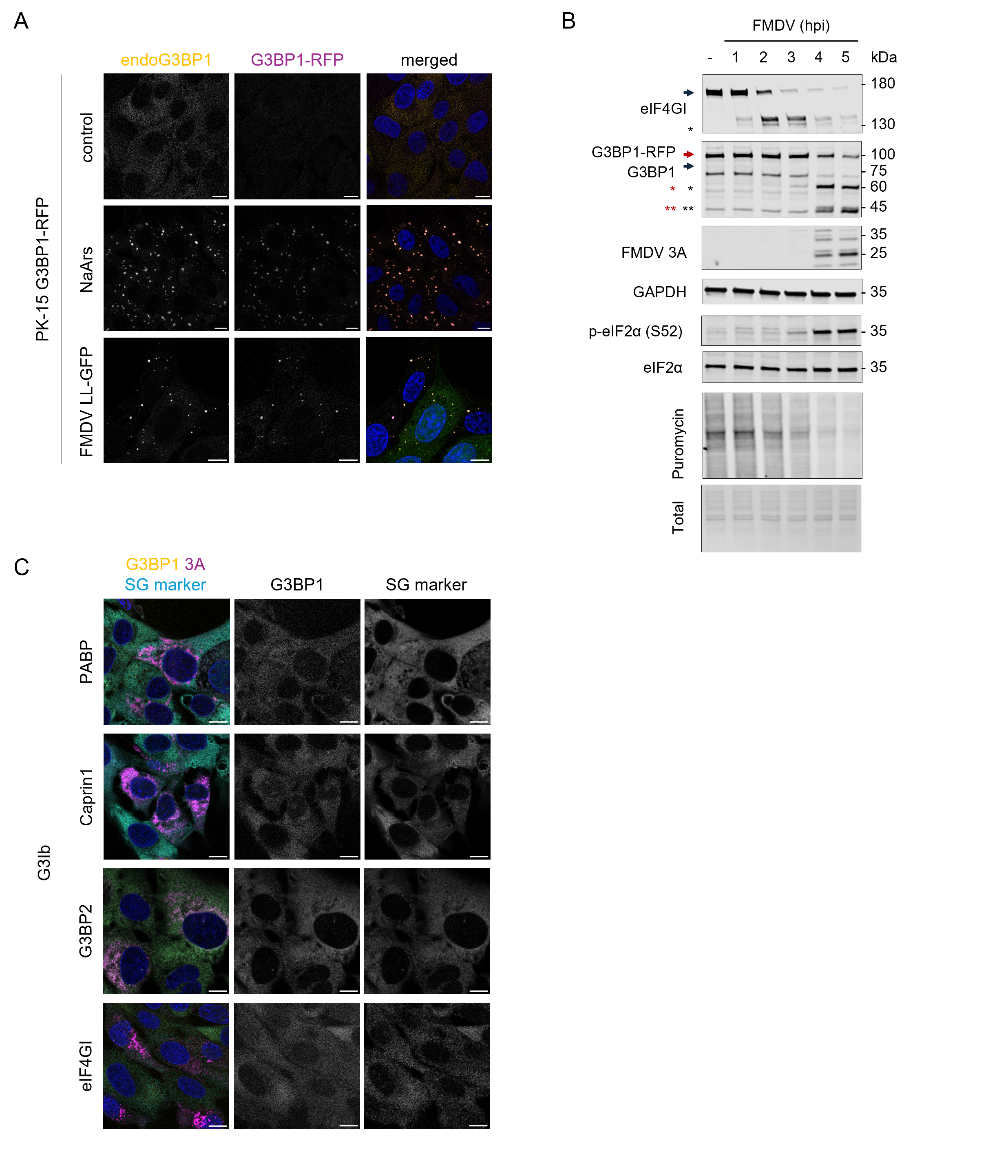

Supplement: S3 Fig — (A) PK-15 G3BP1-RFP cells were treated with sodium arsenite (1 mM for 1 h) or infected with LL-FMDV for 3.5 hours. Cells were analysed by immunofluorescence for the endogenous G3BP1 (gold) and G3BP1-RFP (magenta). Nuclei were stained with DAPI. Scale bars represent 10 μm. (B) Representative western blot for eIF4GI and G3BP1 cleavage, viral infection (FMDV 3A), and integrated stress response activation (phosphorylation of eIF2α and puromycin). Molecular weights are indicated on the right. (C) PK-15 cells were infected with FMDV for 3.5 hours following treatment with G3Ib. Cells were analysed by immunofluorescence for FMDV 3A (magenta), G3BP1 (gold) and a second SG marker, including PABP, caprin1, G3BP2 and eIF4GI (cyan). Nuclei were stained with DAPI. Scale bars represent 10 μm. (TIF) [file ppat.1013722.s003.tif]

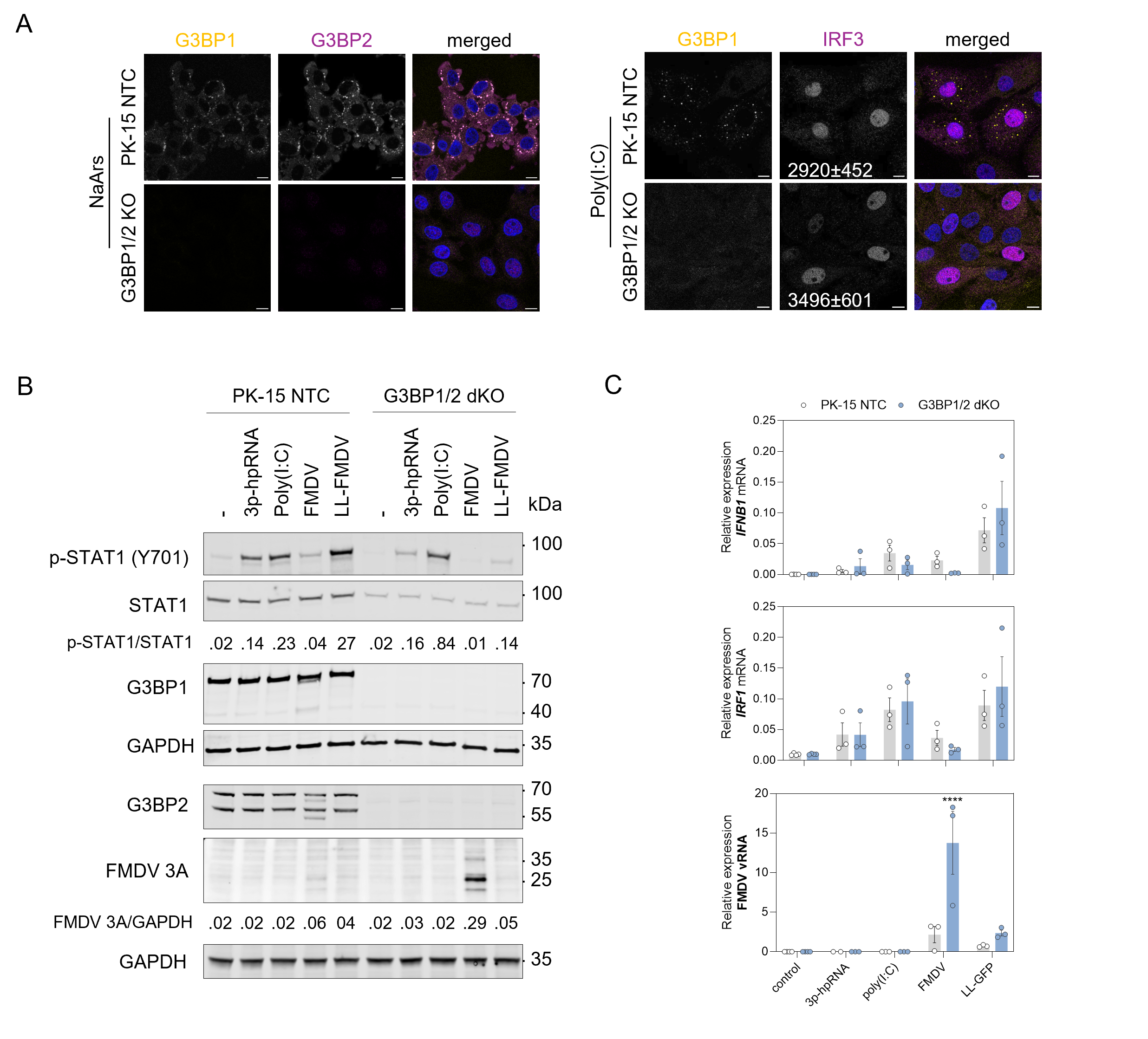

Supplement: S4 Fig — (A) PK-15 NTC or G3BP1/2 KO cells were treated with 1 mM sodium arsenite for 1 h or stimulated with 500 ng poly(I:C) for 4 h. Cells were analysed by immunofluorescence for the SG markers G3BP1 (gold) and G3BP2 or the innate immune response activation markers IRF3 (magenta). Nuclei were stained with DAPI. Scale bars represent 10 μm. (B-C) PK-15 NTC or G3BP1/2 KO cells were transfected with 3p-hpRNA or poly(I:C), or infected with FMDV or LL-FMDV up to 6 hours. (B) Representative immunoblotting of innate immune response activation (STAT1 phosphorylation) and viral protein levels (FMDV 3A). Molecular weights are indicated on the right. Band intensities were normalised to that of total protein or GAPDH. (C) Expression levels of IFNB1 and ISGs were analysed by RT-qPCR with results shown as mean ± SEM, n=3, normalised to GAPDH mRNA. ****p<0.0001; using two-way ANOVA with Šidák’s multiple comparison post-test. (TIF) [file ppat.1013722.s004.tif]

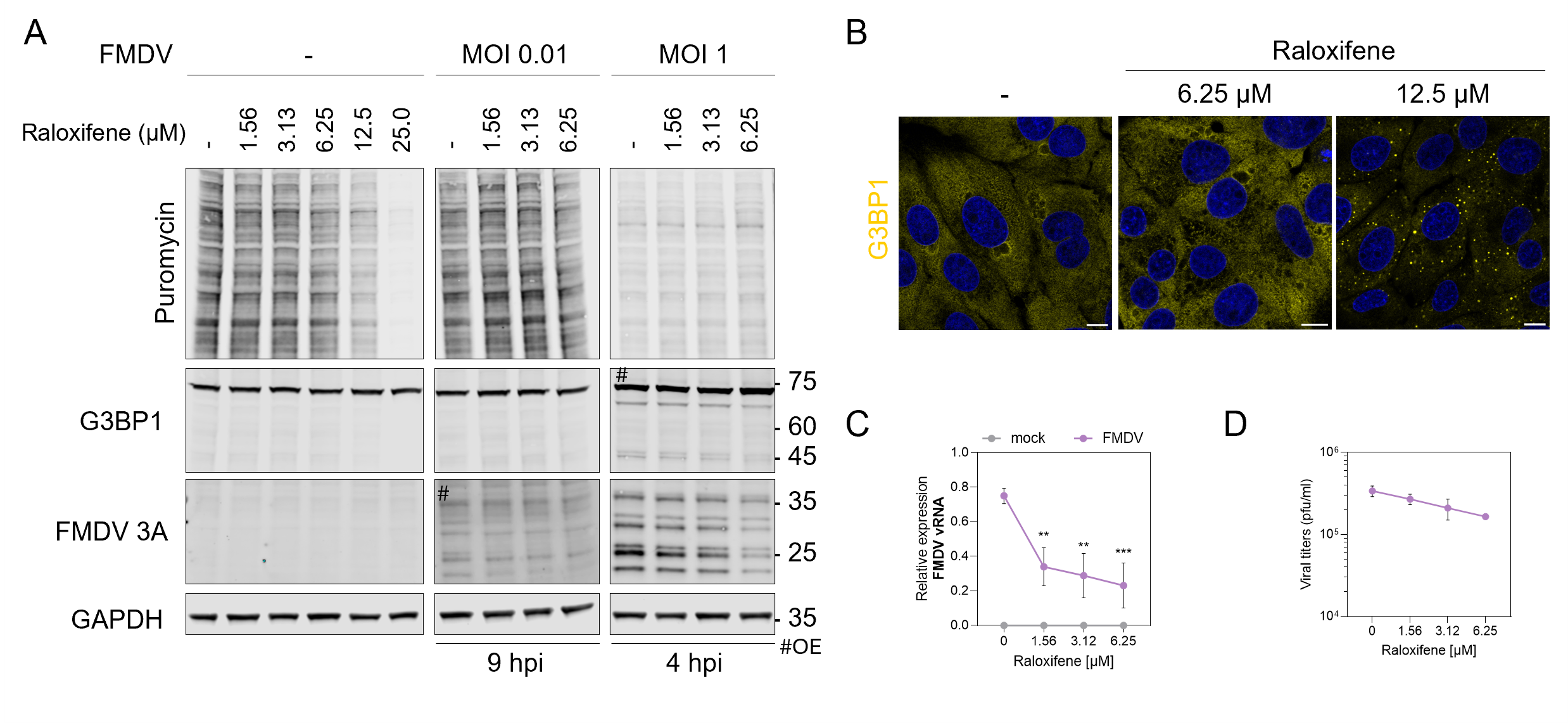

Supplement: S5 Fig — PK-15 cells were treated with increasing concentrations of raloxifene and infected with FMDV at a low or high MOI for the indicated times. (A) Representative western blot for puromycin, indicating translation levels, G3BP1 cleavage and viral infection (FMDV 3A). Molecular weights are indicated on the right. (B) Cells treated with 6.25 or 12.5 μM of raloxifene were analysed by immunofluorescence for the SG marker G3BP1 (gold). Nuclei were stained with DAPI. Scale bars represent 10 μm. (C-D) PK-15 cells were treated with increasing amounts of raloxifene and infected with FMDV at a MOI of 0.01. (C) Expression levels of FMDV genome were analysed by qPCR with results shown as mean ± SEM, n=3, normalised to GAPDH mRNA. (D) FMDV titration at 6 hours post-infection following the treatment with increasing concentrations of raloxifene. Data represents the average of three independent replicates. **p<0.1, *** p<0.001, using two-way ANOVA with Šidák’s multiple comparison between mock-infected or FMDV-infected cells treated with raloxifene and FMDV-infected cells. (TIF) [file ppat.1013722.s005.tif]

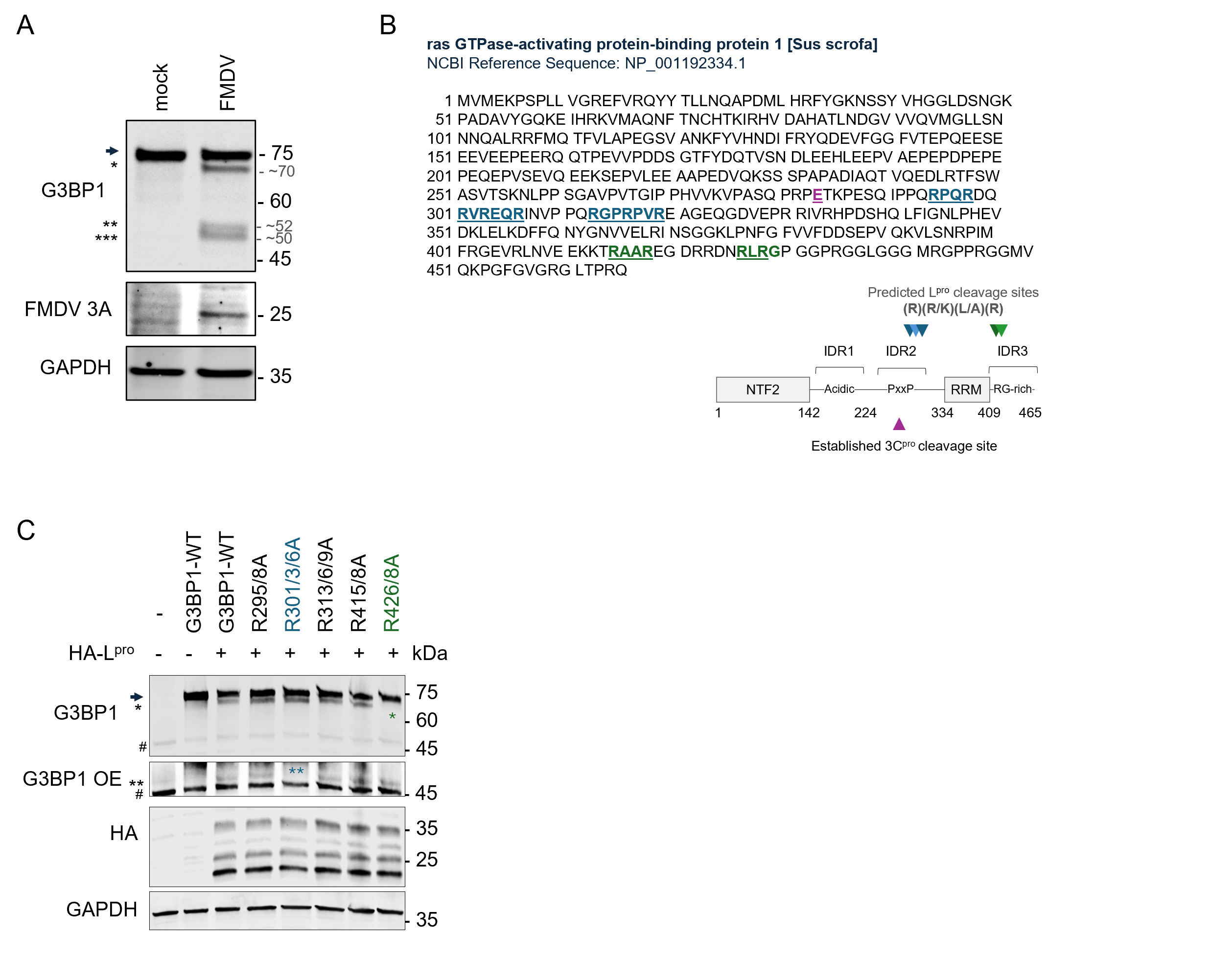

Supplement: S6 Fig — (A) Representative western blot showing G3BP1 cleavage upon FMDV infection. Molecular weights of full length G3BP1 and cleavage products are indicated on the right (*, ** and ***). (B) Available sequence of porcine G3BP1 from NCBI (NP_001192334.1) and representative structure cartoon, highlighting the cleavage site established for 3Cpro (in pink) and the predicted sites for Lpro (in blue and greed). (C) Representative western blot of G3BP1 cleavage by Lpro following co-transfection of viral Lpro and mutated G3BP1 constructs. Molecular weights are indicated on the right and cleavage products (* and **) or unspecific band (#) are indicated on the right. When missing, the cleavage products are represented with asterisks (* or **) with the correspondent colour to the mutations (blue or green). (TIF) [file ppat.1013722.s006.tif]
