# Supplementary material for: The assembly of stress granules during foot-and-mouth disease virus infection is uncoupled from activation of cellular intrinsic antiviral signalling
Source: PLoS Pathog. 2026 Jun 11;22(6):e1013722. doi: 10.1371/journal.ppat.1013722 (PMC13274924; doi:10.1371/journal.ppat.1013722)
Supplement: Attachment — Submitted filename: Response to Reviewers.pdf [file ppat.1013722.s007.pdf]

THE Pirbright INSTITUTE

Professor Nicolas Locker, Ph.D.
Group Leader, Professor of Virology
Pirbright Institute, Pirbright, Woking
Viral Stress Responses | The Pirbright Institute

April 24th, 2026

Dear Prof McInerney,

I am pleased to resubmit for publication our revised manuscript PPATHOGENS-D-25-02855 'The assembly of stress granules during foot-and-mouth disease virus infection is uncoupled from activation of cellular intrinsic antiviral signalling' to PLOS Pathogens.
Following the initial reviews and our additional comments, we performed a significant amount of novel experimental work in particular to fully characterise the role of FMDV protease in stress granules regulation and bolster other aspects of our study as required.
All comments from the 3 reviewers have been answered and addressed, as detailed below, and we hope you will find this updated manuscript now suitable for publication. Of note, given the additional work performed a co-author has been added to the authors list.

Sincerely yours,
Prof Nicolas Locker

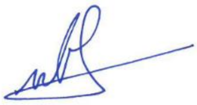

Editor additional comments:
The reviewers agreed that observations regarding the exclusion of a role for SGs in innate immune response were compelling but were less convinced by the experiments with SG assembly and disassembly inhibitors. Consolidation of figures 1-3 into a single figure, as requested by Reviewer 3 might be considered as well as inclusion of extensive analyses to address other points by all 3 reviewers.
We acknowledge all comments, and answered each in turn as detailed below. Overall, we added a significant amount of new data, in particular fully dissecting the impact of the $3C^{pro}$ and L^{pro} on the proteolytic cleavage of G3BP1 and stress granules regulation, and the impact of disassembly inhibitors. As suggested the first figures were consolidated and are now one single supplementary figure. Therefore, we are confident we addressed all the reviewers and editor comments requested.

Part II – Major Issues: Key Experiments Required for Acceptance
Reviewer #1:
#1. Conclusion that blocking SG disassembly has antiviral effect is not well supported by the data. Raloxifene can have other antiviral effects besides SG regulation. In other infection models that authors cite, antiviral effect of raloxifene were not linked to SGs. In their own dose response analysis, authors conclude that at 12.5 micromolar dose raloxifene is causing very strong translation inhibition and SG formation in porcine cells. They chose only 2 times lower dose for virus infection experiments, which still may significantly impair protein synthesis or other metabolic pathways in cells. A different

UKRI Biotechnology and Biological Sciences Research Council
A company limited by guarantee, registered in England no.559784.
The Pirbright Institute is also a registered charity.
The Institute receives strategic funding from BBSRC UKRI.

method to block SG dissolution is required to support the main conclusion. Perhaps, authors could express cleavage-resistant G3BP1 construct as was done in https://pubmed.ncbi.nlm.nih.gov/18005751/.

We acknowledge the lack of understanding for the exact mechanism when using raloxifene to demonstrate the contribution of SGs at late stages in FMDV infection. Yet, we are confident that the observed impairment in SG dissolution is related to stalled FMDV replication and delay in virus-induced G3BP1 cleavage. Regarding the choice of dose treatment: we only observe a clear effect on virus-induced G3BP1 cleavage and virus replication impairment when using 6.25 µM. Concentrations higher than 12.5 µM impact SG formation and impair translation at basal levels however using 6.25 µM, we observe no effect on both mechanisms (Fig. S5). By titrating raloxifene from 1.56 µM to 6.25 µM we show a small effect on viral replication becoming more important at 6.25 µM (Fig. 6D-F), the dose at which we observe a reduction in one of the G3BP1 cleavage products, but not the L^{pro}-dependent eIF4GI cleavage (Fig. 6D). A time-course of FMDV infection in the presence of raloxifene further showed a delay in both viral replication and virus-mediated G3BP1 cleavage supporting our previous observations (Fig. 6G-H and lines 488-505).

To explore an alternative method as suggested, we further characterized G3BP1 cleavage by $3C^{pro}$ and L^{pro}. First, we confirmed the $3C^{pro}$ cleavage site at the previously proposed residue E284 and further mapped the cleavage sites for L^{pro} onto residues R301/3/6 and R426/8 (Fig. 7A-B and S6). We further showed that $3C^{pro}$-mediated cleavage is more important for SG disassembly than L^{pro}-mediated cleavage by using catalytically inactive proteases (Fig. 7C-E). By further performing infection experiments in cells bearing a G3BP1 E284A mutation, we show that SGs become persistent when $3C^{pro}$-mediated cleavage is impaired, suggesting that $3C^{pro}$ regulates SG disassembly (see lines 513-564 and 653-658).

#2. Raloxifene effect on SG disassembly was linked to blocking autophagic flux. FMDV and other picornaviruses induce autophagy and blocking autophagic flux could have negative effects on virus replication. Authors should examine if autophagy is increased in their model and if it is affected by raloxifene treatment. They also should perform plaque assays as they have done in figure 5 and measure viral RNA levels to conclude that virus replication is inhibited by slowing down SG disassembly.

As suggested, we monitored autophagic flux during FMDV infection in the presence of raloxifene. Bafilomycin treatment was used as a control for autophagy inhibition. We confirmed the induction of autophagy in FMDV-infected cells by a reduction in the levels of SQSTM1 (p62) and NDP52, as previously reported in 10.1080/15548627.2025.2597460 and 10.1242/jcs.240622. The expression of both proteins remained altered in the presence of raloxifene, suggesting no effect on autophagy at the tested concentrations. Upon infection, autophagy recovered at 6.25 µM concentration; and we would speculate this is due to the impaired replication we observe upon treatment (Rebuttal Figure 1). As suggested, plaque assays and viral RNA genome levels were measured following FMDV infection and raloxifene treatment (Fig. 6E, F and H, and lines 496-505). Both approaches suggest a small decrease in viral replication when slowing down SG disassembly with raloxifene.

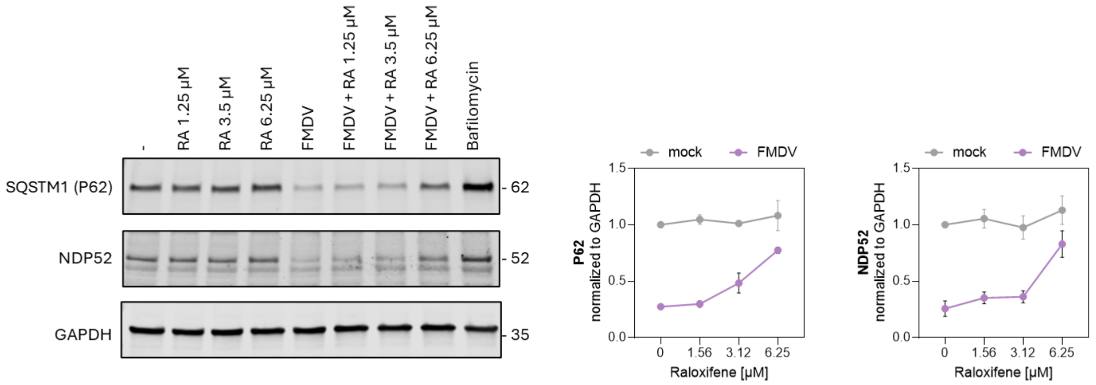

Rebuttal Figure 1: Representative immunoblotting showing SQSTM1 (p62) and NDP52 protein levels following 3.5 hours of FMDV infection in the presence of increasing raloxifene concentrations. Molecular weights are indicated on the right. Band intensities normalised to total protein or GAPDH and represented as mean ± SEM, n=3.

#3. In all infection experiments (figures 4-8) a single SG marker is used (G3BP1). This is insufficient to conclude that SGs disassemble because besides G3BP1 other proteins can form SG (G3BP2, TIA-1). G3BP1 is cleaved by FMDV proteases. Other SG markers should be used to show that SGs disassemble and not just G3BP1 foci disappear.
We have now added other SG markers, including G3BP2, PABP, caprin1 and eIF4GI in our immunofluorescence analysis. As stated in lines 305-307: "All markers, except eIF4GI, colocalize with G3BP1 in SGs during infection with FMDV, suggesting that FMDV-induced granules share components with previously described canonical SGs" (Fig. 2E). We further show that G3Ib promotes the disassembly of G3BP1 and other makers - positive foci following sodium arsenite treatment (lines 209-211, Fig. S2E) or FMDV infection (lines 317-318, Fig. S3C). This overall bolster our results.

Reviewer #2: Major comments:
#1. The formation of stress granules are primarily focused on G3BP-foci...and as such, this should be described as such and as stress granules per se. SGs can be heterogeneous as the authors have stated in the discussion. It would be important to monitor the impact of the condensation/disassembly inhibitors with respect to other stress granule markers such as poly A mRNA, PABP, TIA-1 - standard markers. It is unclear whether the effects or the lack of effects observed are due to G3BP-foci specifically or are SGs in general all affected. This is especially important of the lack of effects by G3Ib...it is possible that other SG marker-foci are still forming.
This is addressed in our answers to 3rd major comment from Reviewer #1, see new Figure 2E and Figures S2E and S3C.

#2. Although there are effects on G3BP-foci formation with inhibitors, what are the levels of G3BP (ex. immunoblotting)? The lack of G3BP-foci could be effects at the protein level.
We observed no change on G3BP1 at protein level when using G3Ib (or its inactive enantiomer), so their effect on SG assembly inhibition is not due to changes in G3BP1 expression (Rebuttal Figure 2).

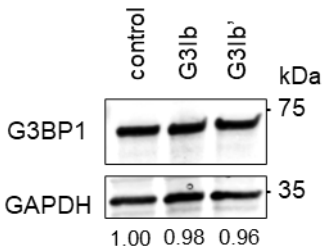

Rebuttal Figure 2: Representative immunoblotting showing G3BP1 protein levels following 3.5 hours of FMDV infection in the presence of G3Ib or G3Ib'. Molecular weights are indicated on the right. Band intensities normalised to total protein or GAPDH.

#3. The condensation/disassembly inhibitors were used to uncouple effects of G3BP-foci formation and innate immune signaling, ISR and FMDV infection. The use of the compounds is unique (the effects of roxafilene have been tested in other virus infections) and have provided alternatives to KD or KO effects. However, it is unclear what the underlying mechanisms are or the specificities of these compounds. For roxafilene, although there are effects on FMDV infection, is this affecting overall protein synthesis (and thus SGs are maintained), RNA replication (ex. RT-qPCR)? What is the specificity of this compound? This is quite important as this is one of the major takeaways from this paper. For G3IB, as above, it will be important to rule out (or in) if there are effects on other SG markers as G3IB did not have an effect on innate immune signaling, FMDV infection. Also the specificity of G3IB is not clear - is this specific to G3BP or could there be off-target effects?
G3Ib has been well characterized and designed to bind to a specific pocket in G3BP1/2 targeted by viral inhibitors of G3BP1/2 function (10.1083/jcb.202308083). Specifically, this modified peptide has been designed based on an 8-mer derived from Chikungunya virus nsp3 that retained the ability to bind the NTF2L domain of G3BP1 to prevent its oligomerization and further nucleation of other SG proteins into SG. The effect on G3BP1 oligomerization was shown not to alter the phosphorylation state of eIF2α or translation rates, to maintain other protein-protein interactions and functions of G3BP1, and to specifically only inhibit its condensation properties. We and others subsequently used G3Ib to drive SG disassembly and study the contribution of SGs to cellular functions (10.1242/jcs.263548). Here, we have shown that G3Ib prevents the accumulation of other SG proteins, such as G3BP2, caprin1, PABP, and eIF4GI (Fig. S2E and S3C). G3Ib is therefore a useful tool that specifically targets G3BP1 and prevents the initial steps of SG assembly, and that allows to us to decouple the effect of SG formation of those of its components in a cellular mechanism, such as the innate immune response.
Raloxifene does not affect the overall protein synthesis at the concentrations tested, on its own, or during infection of FMDV (Fig.S5). This effect was observable upon infection with high MOI as FMDV efficiently blocks translation, yet it was confirmed using a low MOI with no effect on translation rates following raloxifene treatment up to 6.25 µM. RNA replication, measured both by viral genome quantification by RT-qPCR and infectious viral particles by plaque assay (Fig. 6D-F), is slightly affected by raloxifene. This is more evident at 6.25 µM, a point when we observe a reduction in G3BP1 cleavage. A time-course of FMDV infection in the presence of raloxifene showed a delay in both viral replication and virus-mediated G3BP1 cleavage (Fig. 6G-H), supporting our previous observations (lines 488-505). While we lack data on the specificity of raloxifene, it was shown previously to have effects on SG disassembly (dependent on G3BP1) without affecting translation rates (10.1038/s41419-020-03159-5). Furthermore, raloxifene was used before to show an impairment of influenza A virus, SARS-CoV-2, and hepatitis C virus replication (lines 647-650), suggesting a broad mechanism relevant for the life cycle of different viruses. Importantly, these viruses have been shown to modulate SG dynamics (10.1128/JVI.00647-12, 10.1128/jvi.00412-22, 10.1128/JVI.07101-11).

Therefore, we are overall confident in using these compounds to study the impact of SG disassembly and maintenance.

#4. There were several very interesting observations that were not followed up. Fig 2 - the +/- G3BP-foci formation and its effects on IRF1/3 nuclear localization and in Fig 6A, the G3BP-foci formation in bystander cells infected with LL-FMDV. These are super interesting results but there was no cohesive model or further experiments into this (I thought these were the most interesting findings!)

We agree that both findings mentioned are interesting and should be explored further. We are currently pursuing these to explore the role of SGs in bystander cells in driving species-specific outcomes of infection, which will form the basis of an independent manuscript.

The effect on IRF1 and IRF3 nuclear localization is discussed in lines 196-203 and 583-589. We have considered several approaches to unravel this mechanism, *e.g.* FISH analysis of ISG mRNA in these cells or analysis of the different populations by sorting isolation and further characterization. Our main take on these observations is that ISG expression is downregulated in activated cells (nuclear IRF3+) that bear SG and cellular translation is inhibited, but not in the bystander cells stimulated by an interferon-mediated response (nuclear IRF1+) where translation is active.

The formation of G3BP1 foci in bystander cells infected with LL-FMDV (discussed in lines 621-629) is a result we intend to explore further. Understanding the molecular basis of what regulates the bystander effect during infection might help to establish antiviral signalling mechanisms during FMDV infection. We propose this might be related to the activation of a strong antiviral response initiated by to the lack of an active leader protein.

Reviewer #3:

#1. The main point of this manuscript involves the study of SGs assembly in FMDV infected cells and the impact for viral replication in porcine cells. Figures 1-3 show preliminary data regarding the ability of two different viral mimics, poly I:C and 3p-hpRNA, to induce the integrated stress response (ISG) or innate immune response (IIR) and its correlation with the assembly of G3BP1 foci. While these data are interesting, Figures 1-3 can be summarized into a single figure, and most of the 3p-hpRNA results moved to a supplementary figure. In summary, these data show that these viral mimics induce a different viral response in PK15 cells. In contrast to the sentence in l. 148-149, the response to poly I:C treatment is not only more potent but also similar to FMDV infection.

Previous figures 1-3 are now summarized into a single figure and two supplemental figures (Fig. 1 and S1-S2) as suggested and the main text was altered accordingly (from line 129 to 242).

#2. An important question is how G3BP foci are disassembled. Is this solely due to the cleavage of G3BP1, or are there associated factors contributing to this event? Time-course experiment in infected cells shows that G3BP cleavage is a relative late event, compared to eIF4G cleavage (Figure 4). Could it be that the expression of a cleavage-resistant form of G3BP1 does not disassemble SGs? Related to this point, the sentence in l. 484-486 should be modified to cite the work reporting the cleavage of G3BP1 by FMDV 3C (PMID 28755480). This work also showed the interaction of G3BP1 with the FMDV IRES element as well as the IRES-inhibitory activity of this protein. This additional activity of G3BP1 is related to the sentence in l. 427-430 pertaining a role of G3BP1-2 independent from their condensation properties.

We performed further extensive characterization of G3BP1 cleavage by viral proteases (see new Figure 7, and response to Reviewer #1 point 1). We are confident that the cleavage mediated by $3C^{pro}$ on the residue E284 is crucial to initiate SG disassembly, as SGs become persistent during infection in cells bearing a G3BP1 E284A mutation (see lines 513-564).

The text previously on lines 484-486 was modified to include extra results on the effect of raloxifene during infection. The suggested reference (PMID 28755480) is included as part of the discussion (line 642) to support the hypothesis of additional antiviral G3BP1 activities during infection.

#3. Treatment of infected cells with raloxifene (Figure 8 A, B) shows the increase of foci number/cell, as well as the % of cells displaying G3BP1 foci in FMDV infected cells. However, Figure 8D shows

that treatment with raloxifene strongly inhibits G3BP1 cleavage and also viral 3A protein synthesis, but not eIF4G cleavage. Therefore, raloxifene inhibits G3BP1 cleavage, presumably preventing SGs disassembly. The inhibition of 3A synthesis is intriguing as cleavage of eIF4G stimulates IRES-dependent translation, therefore should not hinder 3A synthesis. Comments regarding the action of raloxifene in other viral infections are not informative of raloxifene activities (l. 596-601).
The text has now been changed accordingly (lines 493-495 and 652-657). Treatment of raloxifene inhibits the cleavage of G3BP1 highlighted by a decrease in the smallest cleavage product, but not the highest. We propose that raloxifene does not interfere with L^{pro} proteolytic activity, given similarly it does not modulate eIF4GI cleavage upon infection. Nevertheless, it might interfere with $3C^{pro}$ function, through an unexplored mechanism, that ultimately might help delaying SG disassembly.

Part III – Minor Issues: Editorial and Data Presentation Modifications
Please use this section for editorial suggestions as well as relatively minor modifications of existing data that would enhance clarity.
Reviewer #1:
Figures: throughout the figures, graphs that are stacked vertically have only one x axis labeling on the bottom. The spacing allows for each graph to be labeled, and this will be less confusing.
Figure 1B: dsRNA doses are not indicated. Western blot lanes are not numbered, as in all figures, which makes it difficult to interpret. dsRNA doses are now indicated. We opted not to number lanes to benefit online readers and avoid toggling between the blown-out figures and reaching for details in the legends.
Line 62: IRES is not defined on first use. This was now added in lines 68-69.
Line 139: EIF2AK2 is mentioned without clarifying that this is the gene for PKR which is prominent in the manuscript throughout. This was now added in line 131.
Line 180: “stimulated with 1 ug/ml poly(I:C)” – were the cells transfected or poly(I:C) was added to the media? Please clarify. Cells were transfected with poly(I:C) and this is clarified in lines 157, 166, and 427.
Line 224: first time G3BP1/2 is mentioned in relation to double knockout needed to prevent SG formation. The manuscript is written as if G3BP2 is unimportant. In most instances, SGs are called “G3BP1 foci”. This is confusing. Using additional SG markers can clarify the identity of the foci.
We decided to refer to the detected granules as G3BP1-foci as we are using G3BP1 for virtualisation. Only with the subsequent experiments and characterization were we able to tell that the FMDV-induced granules share features with canonical SG, including the presence of other SG components (e.g., G3BP2, caprin1, PABP, and eIF4GI), and the sensitivity to cycloheximide. A change reflected in the title now stating stress granules instead of stress granules-like.

Reviewer #2: Minor
#5. The authors should describe how the immunofluorescence images were counted? Manually or using a software?
The information regarding the analyses of immunofluorescence images was included in each figure legend. This information was added to the Materials and Methods section in lines 782-787.
#6. What was the source of poly IC? Low or high MW versions used? A low-molecular weight poly(I:C) was used to perform the experiments; see line 731.

Reviewer #3:
#1. The assembly of bigger G3BP1 foci in bystander cells compared to leaderless LL-FMDV infected cells (Figure 6) is intriguing; do the authors have any suggestion about why or how is this? In addition, Figure 6E should show a WB image of G3BP1 and eIF4G.
The formation of G3BP1 foci in bystander cells infected with LL-FMDV (discussed in lines 621-629) is a result we are exploring further. Understanding the molecular basis of what regulates the bystander

effect during infection might help to open new avenues to characterise a novel antiviral signalling mechanism. We propose it may be related to the activation of a strong antiviral response due to the lack of an active leader protein.
As suggested, G3BP1 and eIF4GI staining were included in Fig. 4E (previously Fig. 6E).
#2. Figure 7 shows the assembly of SGs under different conditions, but the polysome profile of treated and untreated cycloheximide cells is lacking.
FMDV is a SAPO-4 pathogen - the highest biosecurity level for livestock pathogens - with infection and experimental work performed in high-containment facilities. Unfortunately, not all molecular biology equipment can be made available in these facilities and therefore we cannot perform polysome profiling of FMDV-infected cells as we did for other viruses in lower containment.
#3. Regarding the role of FMDV L protease to contribute to IFN signaling evasion, the manuscript should cite the work reporting the cleavage of MDA5 and LGP2 by the L protease in infected cells (PMID: 32879301; PMID: 29958302). These references are now included (lines 358 and/or 620).
