# Supplementary material for: The assembly of stress granules during foot-and-mouth disease virus infection is uncoupled from activation of cellular intrinsic antiviral signalling
Source: PLoS Pathog. 2026 Jun 11;22(6):e1013722. doi: 10.1371/journal.ppat.1013722 (PMC13274924; doi:10.1371/journal.ppat.1013722)
Supplement: Attachment — Submitted filename: RebuttalCoverLetter_Marques et al_second revision.pdf [file ppat.1013722.s008.pdf]

Professor Nicolas Locker, Ph.D.
Group Leader, Professor of Virology
Pirbright Institute, Pirbright, Woking
Viral Stress Responses | The Pirbright Institute

May 22nd, 2026

Dear Prof McInerney,

I am pleased to resubmit for publication our revised manuscript PPATHOGENS-D-25-02855 'The assembly of stress granules during foot-and-mouth disease virus infection is uncoupled from activation of cellular intrinsic antiviral signalling' to PLOS Pathogens.
Following the second round of reviews and our additional comments, no major points were highlighted with only minor points to address and general comments.
All these have been answered and addressed, as detailed below, and we hope you will find this updated manuscript now suitable for publication.

Sincerely yours,
Prof Nicolas Locker

Part I – Editorial Issues

1) We do not publish any copyright or trademark symbols that usually accompany proprietary names, eg (R), (C), or TM (e.g. next to drug or reagent names). Please remove all instances of trademark/copyright symbols throughout the text, including [Nunc™ Lab-Tek™ and PowerUp™ SYBR] on page[50, 52] line no[791, 826]
This has been removed as suggested.

Part II – Major Issues: Key Experiments Required for Acceptance

Reviewer #1: N/A
Reviewer #2: (No Response)
Reviewer #3: Figure 7B, and l. 550-551: the product with a mobility close to 45 kDa detected in FMDV infected cells, including unG3BP1, is rather striking, and does not confirm the disappearance of the corresponding cleavage product for each mutation. This figure should be revised, or the sentence should be modified. There is no sufficient resolution to separate the bands marked with red and blue asterisks in G3BP1 well from the 45 kDa band present in unG3BP1 well. Furthermore, none of these products are observed in uninfected cells, suggesting that they may be the result from FMDV infection.
We recognize the presence of this extra band close to 45 kDa in this set of experiments following transfection of G3BP1 constructs and FMDV infection. This band was not previously observed when co-expressing G3BP1 with the viral proteins only, or in endogenous G3BP1 during infection. We

Biotechnology and Biological Sciences Research Council
A company limited by guarantee, registered in England no.559784.
The Pirbright Institute is also a registered charity.
The Institute receives strategic funding from BBSRC UKRI.

hypothesise this may result from proteolytic cleavage of G3BP1 mediated by caspase activation, as previously suggested during viral infection, rather than due to the action of a viral protease. A new sentence was added to the main text to reflect this (lines 554-558, reference 48).

Part III – Minor Issues: Editorial and Data Presentation Modifications
Reviewer #1: As suggested in the previous round of reviews, raloxifene can have other antiviral effects besides SG regulation. In other infection models that authors cite, antiviral effect of raloxifene were not linked to SGs. Authors' own data, including new experiments examining viral RNA levels and FMDV infectious titers, clearly show antiviral effect of raloxifene that is not dependent of autophagy or translation inhibition. However, the delayed disassembly of SGs in infected cells treated with raloxifene only correlates with virus inhibition. It is difficult to separate cause and effect in these experiments because, as authors state in their response, inhibition of FMDV replication causes delay in 3C protease accumulation that in turn delays G3BP1 cleavage that in turn delays SG disassembly. Therefore, inhibition of FMDV replication by raloxifene could be completely SG-independent, regardless of how this drug affects SG dissolution.
The effects of raloxifene on SG disassembly was observed previously during hypoxia in a process that requires G3BP1 (Raloxifene prevents stress granule dissolution, impairs translational control and promotes cell death during hypoxia in glioblastoma cells - PubMed). The effect of raloxifene on SG dissolution during infection was not addressed or not within the scope of the cited research papers. The raloxifene-mediated delay on SG dissolution and potential antiviral role were independent observations, and we cannot take a general conclusion for the raloxifene mode of action out of these. Accordingly, we agree with the complexity in separating causes and effects in these experiments and, therefore, we have changed the statements as suggested by the reviewer in the main abstract (lines 39-42), results (lines 503-512) and discussion (lines 694-695).

Authors chose not to perform experiments testing effects of cleavage-resistant G3BP1 expression on virus replication, even though they had all necessary reagents and cell lines in place and have nicely demonstrated that the mutant is not cleaved in virus-infected cells. Therefore, to be acceptable for publication in PLoS Pathogens, the manuscript has to be modified such that the statements that delaying SG disassembly inhibits FMDV replication and that blocking SG disassembly is a potential therapeutic approach against FMDV should be removed or qualified with more extensive discussion on alternative mechanisms as described above.
While this reviewer states we 'chose not to perform experiments testing effects of cleavage-resistant G3BP1 expression on viral replication, …' we made significant efforts in characterising cleavage products and the impact of protease inactivation and target sites inactivation on stress granules assembly. We would like to stress again that experiments performed in SAPO-4 containment with high-consequence pathogens like FMDV are not trivial and we focussed on essential experiments to answer the comments raised at the time of the initial reviews. We do acknowledge this reviewer comment however and have altered both abstract, results and discussion to temper our conclusion on blocking SG disassembly as therapeutic approach (lines 39-42, lines 503-512, lines 694-695).

Reviewer #2: (No Response)
Reviewer #3:
L. 286 is lacking a reference for host shut off cleavage associated with eIF4G cleavage
References added (lines 287).

L 413. Please clarify this sentence: cycloheximide treatment inhibits translation elongation, preventing the assembly of canonical SGs, among many other processes.
Sentence rephrased accordingly (lines 413-416).
