# Supplementary material for: The assembly of stress granules during foot-and-mouth disease virus infection is uncoupled from activation of cellular intrinsic antiviral signalling
Source: PLoS Pathog. 2026 Jun 11;22(6):e1013722. doi: 10.1371/journal.ppat.1013722 (PMC13274924; doi:10.1371/journal.ppat.1013722)
Supplement: Peer review history

# Peer review: Decision Letter 0

**DOI:** 10.1371/journal.ppat.1013722.r001

**Authors:** Alexander E. Gorbalenya, Gerald M McInerney

21 Dec 2025

-->PPATHOGENS-D-25-02855

The assembly of stress granule-like foci during foot-and-mouth disease virus infection is uncoupled from activation of cellular intrinsic antiviral signalling

PLOS Pathogens

Dear Dr. Locker,

Thank you for submitting your manuscript to PLOS Pathogens. After careful consideration, we feel that it has merit but does not fully meet PLOS Pathogens's publication criteria as it currently stands. Therefore, we invite you to submit a revised version of the manuscript that addresses the points raised during the review process.

In particular, the reviewers agreed that observations regarding the exclusion of a role for SGs in innate immune response were compelling, but were less convinced by the experiments with SG assembly and disassembly inhibitors. Consolidation of figures 1-3 into a single figure, as requested by Reviewer 3 might be considered as well as inclusion of extensive analyses to address other points by all 3 reviewers.

Please submit your revised manuscript by March 11th, 2026. If you will need more time than this to complete your revisions, please reply to this message or contact the journal office at plospathogens@plos.org. When you're ready to submit your revision, log on to https://www.editorialmanager.com/ppathogens/ and select the 'Submissions Needing Revision' folder to locate your manuscript file.

Please include the following items when submitting your revised manuscript:

* A letter that responds to each point raised by the editor and reviewer(s). You should upload this letter as a separate file labeled 'Response to Reviewers'. This file does not need to include responses to any formatting updates and technical items listed in the 'Journal Requirements' section below.

* A marked-up copy of your manuscript that highlights changes made to the original version. You should upload this as a separate file labeled 'Revised Manuscript with Track Changes'.

* An unmarked version of your revised paper without tracked changes. You should upload this as a separate file labeled 'Manuscript'.

If you would like to make changes to your financial disclosure, competing interests statement, or data availability statement, please make these updates within the submission form at the time of resubmission. Guidelines for resubmitting your figure files are available below the reviewer comments at the end of this letter.

We look forward to receiving your revised manuscript.

Kind regards,

Gerald M McInerney

Academic Editor

PLOS Pathogens

Alexander Gorbalenya

Section Editor

PLOS Pathogens

Sumita Bhaduri-McIntosh

Editor-in-Chief

PLOS Pathogens

orcid.org/0000-0003-2946-9497

Michael Malim

-->Editor-in-Chief

PLOS Pathogens

orcid.org/0000-0002-7699-2064

**Additional Editor Comments:**

In particular, the reviewers agreed that observations regarding the exclusion of a role for SGs in innate immune response were compelling, but were less convinced by the experiments with SG assembly and disassembly inhibitors. Consolidation of figures 1-3 into a single figure, as requested by Reviewer 3 might be considered as well as inclusion of extensive analyses to address other points by all 3 reviewers.

**Journal Requirements:**

If the reviewer comments include a recommendation to cite specific previously published works, please review and evaluate these publications to determine whether they are relevant and should be cited. There is no requirement to cite these works unless the editor has indicated otherwise.

1) We ask that a manuscript source file is provided at Revision. Please upload your manuscript file as a .doc, .docx, .rtf or .tex. If you are providing a .tex file, please upload it under the item type u2018LaTeX Source Fileu2019 and leave your .pdf version as the item type u2018Manuscriptu2019.

2) We do not publish any copyright or trademark symbols that usually accompany proprietary names, eg ©,  ®, or TM  (e.g. next to drug or reagent names). Therefore please remove all instances of trademark/copyright symbols throughout the text, including:

- TM on page: 30.

**Reviewers' Comments:**

Reviewer's Responses to Questions

**Part I - Summary**

Please use this section to discuss strengths/weaknesses of study, novelty/significance, general execution and scholarship.

Reviewer #1: Manuscript “The assembly of stress granule-like foci during foot-and-mouth disease virus infection is uncoupled from activation of cellular intrinsic antiviral signalling” by Marques et al. analyzes SG formation in porcine cells treated with dsRNA ligands or infected with FMDV and how they influence innate immune responses and virus replication. They show that the formation of SG following a challenge with dsRNA mimic poly(I:C) does not modulate the activation of innate immune response as measured by induction of interferon and ISGs by qPCR and western blot. In FMDV infection, they observe transient assembly of SG followed by virus-induced cleavage of G3BP1 and SG disassembly. When SG formation in infected cells was inhibited by a small molecule G3Ib, viral replication and antiviral response were not impacted, arguing against the hypothesis that SGs potentiate antiviral signaling. Treatment of infected cells with raloxifene, a selective estrogen receptor modulator (SERM) drug that was reported to delay SG disassembly in the model of hypoxia, decreased viral protein accumulation and virus-induced cell death. Based on these results, authors suggest that SG assembly does not impact viral replication or antiviral response, but preventing SG disassembly negatively impacts FMDV replication, making disassembly a potential therapeutic target to prevent FMDV infection. The manuscript focuses on an important and somewhat controversial topic of innate immunity-regulating and antiviral functions of SG. The data supporting the conclusion that SGs do not increase innate immune response is convincing and worth reporting. However, the main conclusion that blocking SG dissolution inhibits viral replication is not sufficiently supported.

Reviewer #2: The manuscript by Marques et al. examines the role of G3BP-foci in integrated stress response, translation status and innate immune signaling during poly IC and FMDV infection. Although studied extensively, the roles of stress granules in antiviral innate immune responses and virus infection are not fully understood - partly because SGs may have pleiotropic functions and are form due to translational initiation shutoff. Uncoupling these effects have been a challenge in the field. In this study, the authors have uncoupled some of these roles by using specific reagents and compounds. The authors have shown (highlights):

1. Poly IC treatment leads to induction of phospho-eIF2alpha, phospho-IRF3, induction of ISGs, and induced G3BP-foci whereas a RIG-I/MDA5 specific inducer did not, suggesting that RIG-I signaling does not specifically induce the ISR and SG formation.

2. Poly IC induced G3BP-foci formation - in cells with G3BP-foci formation, it did not lead to IRF1/3 nuclear translocation.

3. Using a G3Ib condensation inhibitor, blocking G3BP SG formation had no effect on poly IC induction of ISG, ISR signaling and no impact on FMDV infection and on innate immune signaling in FMDV-infected cells, suggesting that G3BP-foci SG formation does not promote downstream signaling.

4. Use of raloxifene, which reduces disassembly of SGs, there is a modest attenuation of FMDV infection (MOI 0.1) but not on ISR signaling, host shutoff.

The authors have used a number of reagents in this study to uncouple and clarify the effects of G3BP-SG formation. Overall, most of the experiments are well-designed and clear. However, some results are open to alternative interpretation, especially on the specificities of the condensation and disassembly inhibitors. The study is a bit of a mix of experiments and results with no cohesive conclusion...there are definitely several results that are interesting and informative (timing of SG formation, relationship to protein synthesis) and clarifies the role (or lack of) of SGs on ISR and innate immune signaling, which are important findings for the field. These findings suggest that G3BP-foci SGs may indeed be a consequence and not a promoter of downstream effects (at least with the signaling pathways and FMDV infection in this cell line assayed). However, the underlying mechanisms are not understood and there is a lack of a unifying model.

Reviewer #3: This manuscript reports the assembly of cytoplasmic stress granules (SGs) during FMDV infection followed by virus-induced cleavage of G3BP1. Authors claim that assembly of SGs does not affect viral replication or antiviral response, while inhibition of their disassembly using raloxifene negatively impacts FMDV replication. The manuscript is well written. However, several concerns need to be addressed prior to publication.

**********

**Part II – Major Issues: Key Experiments Required for Acceptance**

Please use this section to detail the key new experiments or modifications of existing experiments that should be absolutely required to validate study conclusions.

Generally, there should be no more than 3 such required experiments or major modifications for a "Major Revision" recommendation. If more than 3 experiments are necessary to validate the study conclusions, then you are encouraged to recommend "Reject".

Reviewer #1: 1. Conclusion that blocking SG disassembly has antiviral effect is not well supported by the data. Raloxifene can have other antiviral effects besides SG regulation. In other infection models that authors cite, antiviral effect of raloxifene were not linked to SGs. In their own dose response analysis, authors conclude that at 12.5 micromolar dose raloxifene is causing very strong translation inhibition and SG formation in porcine cells. They chose only 2 times lower dose for virus infection experiments, which still may significantly impair protein synthesis or other metabolic pathways in cells. A different method to block SG dissolution is required to support the main conclusion. Perhaps, authors could express cleavage-resistant G3BP1 construct as was done in https://pubmed.ncbi.nlm.nih.gov/18005751/.

2. Raloxifene effect on SG disassembly was linked to blocking autophagic flux. FMDV and other picornaviruses induce autophagy and blocking autophagic flux could have negative effects on virus replication. Authors should examine if autophagy is increased in their model and if it is affected by raloxifene treatment. They also should perform plaque assays as they have done in figure 5 and measure viral RNA levels to conclude that virus replication is inhibited by slowing down SG disassembly.

3. In all infection experiments (figures 4-8) a single SG marker is used (G3BP1). This is insufficient to conclude that SGs disassemble because besides G3BP1 other proteins can form SG (G3BP2, TIA-1). G3BP1 is cleaved by FMDV proteases. Other SG markers should be used to show that SGs disassemble and not just G3BP1 foci disappear.

Reviewer #2: Major comments:

1. The formation of stress granules are primarily focused on G3BP-foci...and as such, this should be described as such and as stress granules per se. SGs can be heterogeneous as the authors have stated in the discussion. It would be important to monitor the impact of the condensation/disassembly inhibitors with respect to other stress granule markers such as poly A mRNA, PABP, TIA-1 - standard markers. It is unclear whether the effects or the lack of effects observed are due to G3BP-foci specifically or are SGs in general all affected. This is especially important of the lack of effects by G3lb...it is possible that other SG marker-foci are still forming.

2. Although there are effects on G3BP-foci formation with inhibitors, what are the levels of G3BP (ex. immunoblotting)? The lack of G3BP-foci could be effects at the protein level.

3. The condensation/disassembly inhibitors were used to uncouple effects of G3BP-foci formation and innate immune signaling, ISR and FMDV infection. The use of the compounds is unique (the effects of roxafilene have been tested in other virus infections) and have provided alternatives to KD or KO effects. However, it is unclear what the underlying mechanisms are or the specificities of these compounds. For roxafilene, although there are effects on FMDV infection, is this affecting overall protein synthesis (and thus SGs are maintained), RNA replication (ex. RT-qPCR)? What is the specificity of this compound? This is quite important as this is one of the major takeaways from this paper. For G3lB, as above, it will be important to rule out (or in) if there are effects on other SG markers as G3lB did not have an effect on innate immune signaling, FMDV infection. Also the specificity of G3lB is not clear - is this specific to G3BP or could there be off-target effects?

4. There were several very interesting observations that were not followed up. Fig 2 - the +/- G3BP-foci formation and its effects on IRF1/3 nuclear localization and in Fig 6A, the G3BP-foci formation in bystander cells infected with LL-FMDV. These are super interesting results but there was no cohesive model or further experiments into this (I thought these were the most interesting findings!)

Reviewer #3: 1.The main point of this manuscript involves the study of SGs assembly in FMDV infected cells and the impact for viral replication in porcine cells. Figures 1-3 show preliminary data regarding the ability of two different viral mimics, poly I:C and 3p-hpRNA, to induce the integrated stress response (ISG) or innate immune response (IIR) and its correlation with the assembly of G3BP1 foci. While these data are interesting, Figures 1-3 can be summarized into a single figure, and most of the 3p-hpRNA results moved to a supplementary figure. In summary, these data show that these viral mimics induce a different viral response in PK15 cells. In contrast to the sentence in l. 148-149, the response to poly I:C treatment is not only more potent but also similar to FMDV infection.

2.An important question is how G3BP foci are disassembled. Is this solely due to the cleavage of G3BP1, or are there associated factors contributing to this event? Time-course experiment in infected cells shows that G3BP cleavage is a relative late event, compared to eIF4G cleavage (Figure 4). Could it be that the expression of a cleavage-resistant form of G3BP1 does not disassemble SGs? Related to this point, the sentence in l. 484-486 should be modified to cite the work reporting the cleavage of G3BP1 by FMDV 3C (PMID 28755480). This work also showed the interaction of G3BP1 with the FMDV IRES element as well as the IRES-inhibitory activity of this protein. This additional activity of G3BP1 is related to the sentence in l. 427-430 pertaining a role of G3BP1-2 independent from their condensation properties.

3. Treatment of infected cells with raloxifene (Figure 8 A, B) shows the increase of foci number/cell, as well as the % of cells displaying G3BP1 foci in FMDV infected cells. However, Figure 8D shows that treatment with raloxifene strongly inhibits G3BP1 cleavage and also viral 3A protein synthesis, but not eIF4G cleavage. Therefore, raloxifene inhibits G3BP1 cleavage, presumably preventing SGs disassembly. The inhibition of 3A synthesis is intriguing as cleavage of eIF4G stimulates IRES-dependent translation, therefore should not hinder 3A synthesis. Comments regarding the action of raloxifene in other viral infections are not informative of raloxifene activities (l. 596-601).

**********

**Part III – Minor Issues: Editorial and Data Presentation Modifications**

Please use this section for editorial suggestions as well as relatively minor modifications of existing data that would enhance clarity.

Reviewer #1: Figures: throughout the figures, graphs that are stacked vertically have only one x axis labeling on the bottom. The spacing allows for each graph to be labeled, and this will be less confusing.

Figure 1B: dsRNA doses are not indicated. Western blot lanes are not numbered, as in all figures, which makes it difficult to interpret.

Line 62: IRES is not defined on first use.

Line 139: EIF2AK2 is mentioned without clarifying that this is the gene for PKR which is prominent in the manuscript throughout.

Line 180: “stimulated with 1 ug/ml poly(I:C)” – were the cells transfected or poly(I:C) was added to the media? Please clarify.

Line 224: first time G3BP1/2 is mentioned in relation to double knockout needed to prevent SG formation. The manuscript is written as if G3BP2 is unimportant. In most instances, SGs are called “G3BP1 foci”. This is confusing. Using additional SG markers can clarify the identity of the foci.

Reviewer #2: Minor

5. The authors should describe how the immunofluorescence images were counted? Manually or using a software?

6. What was the source of poly IC? Low or high MW versions used?

Reviewer #3: 1. The assembly of bigger G3BP1 foci in bystander cells compared to leaderless LL-FMDV infected cells (Figure 6) is intriguing; do the authors have any suggestion about why or how is this? In addition, Figure 6E should show a WB image of G3BP1 and eIF4G.

2. Figure 7 shows the assembly of SGs under different conditions, but the polysome profile of treated and untreated cycloheximide cells is lacking.

3. Regarding the role of FMDV L protease to contribute to IFN signaling evasion, the manuscript should cite the work reporting the cleavage of MDA5 and LGP2 by the L protease in infected cells (PMID: 32879301; PMID: 29958302).

**********

PLOS authors have the option to publish the peer review history of their article (what does this mean?). If published, this will include your full peer review and any attached files.

If you choose “no”, your identity will remain anonymous but your review may still be made public.

**Do you want your identity to be public for this peer review?**   For information about this choice, including consent withdrawal, please see our Privacy Policy.

Reviewer #1: No

Reviewer #2: No

Reviewer #3: No

**Figure resubmission:**

-->While revising your submission, we strongly recommend that you use PLOS’s NAAS tool (https://ngplosjournals.pagemajik.ai/artanalysis) to test your figure files. NAAS can convert your figure files to the TIFF file type and meet basic requirements (such as print size, resolution), or provide you with a report on issues that do not meet our requirements and that NAAS cannot fix.

After uploading your figures to PLOS’s NAAS tool - https://ngplosjournals.pagemajik.ai/artanalysis, NAAS will process the files provided and display the results in the "Uploaded Files" section of the page as the processing is complete. If the uploaded figures meet our requirements (or NAAS is able to fix the files to meet our requirements), the figure will be marked as "fixed" above. If NAAS is unable to fix the files, a red "failed" label will appear above. When NAAS has confirmed that the figure files meet our requirements, please download the file via the download option, and include these NAAS processed figure files when submitting your revised manuscript.

**Reproducibility:**

To enhance the reproducibility of your results, we recommend that authors of applicable studies deposit laboratory protocols in protocols.io, where a protocol can be assigned its own identifier (DOI) such that it can be cited independently in the future. Additionally, PLOS ONE offers an option to publish peer-reviewed clinical study protocols. Read more information on sharing protocols at https://plos.org/protocols?utm_medium=editorial-email&utm_source=authorletters&utm_campaign=protocols

# Author comment: Author response to Decision Letter 1

**DOI:** 10.1371/journal.ppat.1013722.r002

28 Apr 2026

Attachment Submitted filename: Response to Reviewers.pdf

# Peer review: Decision Letter 1

**DOI:** 10.1371/journal.ppat.1013722.r003

**Authors:** Alexander E. Gorbalenya, Gerald M McInerney

21 May 2026

PPATHOGENS-D-25-02855R1

The assembly of stress granules during foot-and-mouth disease virus infection is uncoupled from activation of cellular intrinsic antiviral signalling

PLOS Pathogens

Dear Dr. Locker,

Thank you for submitting your manuscript to PLOS Pathogens. After careful consideration, we feel that it has merit but does not fully meet PLOS Pathogens's publication criteria as it currently stands. Therefore, we invite you to submit a revised version of the manuscript that addresses the points raised during the review process.

Please submit your revised manuscript by Jul 20 2026 11:59PM. If you will need more time than this to complete your revisions, please reply to this message or contact the journal office at plospathogens@plos.org. When you're ready to submit your revision, log on to https://www.editorialmanager.com/ppathogens/ and select the 'Submissions Needing Revision' folder to locate your manuscript file.

Please include the following items when submitting your revised manuscript:

* A letter that responds to each point raised by the editor and reviewer(s). You should upload this letter as a separate file labeled 'Response to Reviewers'. This file does not need to include responses to any formatting updates and technical items listed in the 'Journal Requirements' section below.

* A marked-up copy of your manuscript that highlights changes made to the original version. You should upload this as a separate file labeled 'Revised Manuscript with Track Changes'.

* An unmarked version of your revised paper without tracked changes. You should upload this as a separate file labeled 'Manuscript'.

If you would like to make changes to your financial disclosure, competing interests statement, or data availability statement, please make these updates within the submission form at the time of resubmission. Guidelines for resubmitting your figure files are available below the reviewer comments at the end of this letter.

As the corresponding author, your ORCID iD is verified in the submission system and will appear in the published article. PLOS supports the use of ORCID, and we encourage all coauthors to register for an ORCID iD and use it as well. Please encourage your coauthors to verify their ORCID iD within the submission system before final acceptance, as unverified ORCID iDs will not appear in the published article. Only the individual author can complete the verification step; PLOS staff cannot verify ORCID iDs on behalf of authors.

We look forward to receiving your revised manuscript.

Kind regards,

Gerald M McInerney

Academic Editor

PLOS Pathogens

Alexander Gorbalenya

Section Editor

PLOS Pathogens

Sumita Bhaduri-McIntosh

Editor-in-Chief

PLOS Pathogens

orcid.org/0000-0003-2946-9497

Michael Malim

Editor-in-Chief

PLOS Pathogens

orcid.org/0000-0002-7699-2064

**Journal Requirements:**

If the reviewer comments include a recommendation to cite specific previously published works, please review and evaluate these publications to determine whether they are relevant and should be cited. There is no requirement to cite these works unless the editor has indicated otherwise.

1) We do not publish any copyright or trademark symbols that usually accompany proprietary names, eg (R), (C), or TM (e.g. next to drug or reagent names). Please remove all instances of trademark/copyright symbols throughout the text, including [ Nunc™ Lab-Tek™ and  PowerUp™ SYBR] -->on page[ 50, 52] line no[ 79-->1, 826]

**Reviewers' Comments:**

Reviewer's Responses to Questions

**Part I - Summary**

Please use this section to discuss strengths/weaknesses of study, novelty/significance, general execution and scholarship.

Reviewer #1: Manuscript “The assembly of stress granule-like foci during foot-and-mouth disease virus infection is uncoupled from activation of cellular intrinsic antiviral signalling” by Marques et al. analyzes SG formation in porcine cells treated with dsRNA ligands or infected with FMDV and how they influence innate immune responses and virus replication. The manuscript focuses on an important topic of innate immunity-regulating and antiviral functions of SG. The data supporting the conclusion that SGs do not increase innate immune response is convincing. The conclusions are now further boosted in the revised manuscript by inclusion of other SG markers in addition to G3BP1. Authors also characterized G3BP1 cleavage by FMDV 3C protease and provide strong evidence that this cleavage is separate from activities of L protease that has other functions in suppressing antiviral responses. Overall, authors included good amount of new experimental data and addressed most of the previous comments. Unfortunately, the final statement in the abstract about SGs that “manipulating their disassembly limits replication and might serve as a potential therapeutic target to prevent FMDV infection” is still insufficiently supported.

Reviewer #2: The authors have responded to all comments appropriately and/or provided experiments to support their conclusions.

Reviewer #3: The revised manuscript is greatly improved. The authors addressed most of my concerns. In particular, association of G3BP cleavage with SGs disassembly is one of the main conclusions of the manuscript. Yet, the mechanism of action of raloxifene is not understood. On the other hand, the assembly of bigger G3BP foci in bystander cells compared to LL-FMDV remains to be studied in future studies, as stated in the rebuttal letter.

**********

**Part II – Major Issues: Key Experiments Required for Acceptance**

Please use this section to detail the key new experiments or modifications of existing experiments that should be absolutely required to validate study conclusions.

Generally, there should be no more than 3 such required experiments or major modifications for a "Major Revision" recommendation. If more than 3 experiments are necessary to validate the study conclusions, then you are encouraged to recommend "Reject".

Reviewer #1: N/A

Reviewer #2: (No Response)

Reviewer #3: Figure 7B, and l. 550-551: the product with a mobility close to 45 kDa detected in FMDV infected cells, including unG3BP1, is rather striking, and does not confirm the disappearance of the corresponding cleavage product for each mutation. This figure should be revised, or the sentence should be modified. There is no sufficient resolution to separate the bands marked with red and blue asterisks in G3BP1 well from the 45 kDa band present in unG3BP1 well. Furthermore, none of these products are observed in uninfected cells, suggesting that they may be the result from FMDV infection.

**********

**Part III – Minor Issues: Editorial and Data Presentation Modifications**

Please use this section for editorial suggestions as well as relatively minor modifications of existing data that would enhance clarity.

Reviewer #1: As suggested in the previous round of reviews, raloxifene can have other antiviral effects besides SG regulation. In other infection models that authors cite, antiviral effect of raloxifene were not linked to SGs. Authors’ own data, including new experiments examining viral RNA levels and FMDV infectious titers, clearly show antiviral effect of raloxifene that is not dependent of autophagy or translation inhibition. However, the delayed disassembly of SGs in infected cells treated with raloxifene only correlates with virus inhibition. It is difficult to separate cause and effect in these experiments because, as authors state in their response, inhibition of FMDV replication causes delay in 3C protease accumulation that in turn delays G3BP1 cleavage that in turn delays SG disassembly. Therefore, inhibition of FMDV replication by raloxifene could be completely SG-independent, regardless of how this drug affects SG dissolution. Authors chose not to perform experiments testing effects of cleavage-resistant G3BP1 expression on virus replication, even though they had all necessary reagents and cell lines in place and have nicely demonstrated that the mutant is not cleaved in virus-infected cells. Therefore, to be acceptable for publication in PLoS Pathogens, the manuscript has to be modified such that the statements that delaying SG disassembly inhibits FMDV replication and that blocking SG disassembly is a potential therapeutic approach against FMDV should be removed or qualified with more extensive discussion on alternative mechanisms as described above.

Reviewer #2: (No Response)

Reviewer #3: L. 286 is lacking a reference for host shut off cleavage associated with eIF4G cleavage

L 413. Please clarify this sentence: cycloheximide treatment inhibits translation elongation, preventing the assembly of canonical SGs, among many other processes.

**********

PLOS authors have the option to publish the peer review history of their article (what does this mean?). If published, this will include your full peer review and any attached files.

If you choose “no”, your identity will remain anonymous but your review may still be made public.

**Do you want your identity to be public for this peer review?**   For information about this choice, including consent withdrawal, please see our Privacy Policy.

Reviewer #1: No

Reviewer #2: No

Reviewer #3: No

[NOTE: If reviewer comments were submitted as an attachment file, they will be attached to this email and accessible via the submission site. Please log into your account, locate the manuscript record, and check for the action link "View Attachments". If this link does not appear, there are no attachment files.]

**Figure resubmission:**

-->While revising your submission, we strongly recommend that you use PLOS’s NAAS tool (https://ngplosjournals.pagemajik.ai/artanalysis) to test your figure files. NAAS can convert your figure files to the TIFF file type and meet basic requirements (such as print size, resolution), or provide you with a report on issues that do not meet our requirements and that NAAS cannot fix.

After uploading your figures to PLOS’s NAAS tool - https://ngplosjournals.pagemajik.ai/artanalysis, NAAS will process the files provided and display the results in the "Uploaded Files" section of the page as the processing is complete. If the uploaded figures meet our requirements (or NAAS is able to fix the files to meet our requirements), the figure will be marked as "fixed" above. If NAAS is unable to fix the files, a red "failed" label will appear above. When NAAS has confirmed that the figure files meet our requirements, please download the file via the download option, and include these NAAS processed figure files when submitting your revised manuscript.

**Reproducibility:**

To enhance the reproducibility of your results, we recommend that authors of applicable studies deposit laboratory protocols in protocols.io, where a protocol can be assigned its own identifier (DOI) such that it can be cited independently in the future. Additionally, PLOS ONE offers an option to publish peer-reviewed clinical study protocols. Read more information on sharing protocols at https://plos.org/protocols?utm_medium=editorial-email&utm_source=authorletters&utm_campaign=protocols

# Author comment: Author response to Decision Letter 2

**DOI:** 10.1371/journal.ppat.1013722.r004

26 May 2026

Attachment Submitted filename: RebuttalCoverLetter_Marques et al_second revision.pdf

# Editor report: Decision Letter 2

**DOI:** 10.1371/journal.ppat.1013722.r005

**Authors:** Alexander E. Gorbalenya, Gerald M McInerney

29 May 2026

Dear Prof. Locker,

We are pleased to inform you that your manuscript 'The assembly of stress granules during foot-and-mouth disease virus infection is uncoupled from activation of cellular intrinsic antiviral signalling' has been provisionally accepted for publication in PLOS Pathogens.

Before your manuscript can be formally accepted you will need to complete some formatting changes, which you will receive in a follow up email. A member of our team will be in touch with a set of requests.

Please note that your manuscript will not be scheduled for publication until you have made the required changes, so a swift response is appreciated.

IMPORTANT: The editorial review process is now complete. PLOS will only permit corrections to spelling, formatting or significant scientific errors from this point onwards. Requests for major changes, or any which affect the scientific understanding of your work, will cause delays to the publication date of your manuscript.

Should you, your institution's press office or the journal office choose to press release your paper, you will automatically be opted out of early publication. We ask that you notify us now if you or your institution is planning to press release the article. All press must be co-ordinated with PLOS.

Thank you again for supporting Open Access publishing; we are looking forward to publishing your work in PLOS Pathogens.

Best regards,

Gerald M McInerney

Academic Editor

PLOS Pathogens

Alexander Gorbalenya

Section Editor

PLOS Pathogens

Sumita Bhaduri-McIntosh

Editor-in-Chief

PLOS Pathogens

orcid.org/0000-0003-2946-9497

Michael Malim

Editor-in-Chief

PLOS Pathogens

orcid.org/0000-0002-7699-2064

***********************************************************

Reviewer Comments (if any, and for reference):

# Editor report: Acceptance letter

**DOI:** 10.1371/journal.ppat.1013722.r006

**Authors:** Alexander E. Gorbalenya, Gerald M McInerney

Dear Prof. Locker,

We are delighted to inform you that your manuscript, "The assembly of stress granules during foot-and-mouth disease virus infection is uncoupled from activation of cellular intrinsic antiviral signalling," has been formally accepted for publication in PLOS Pathogens.

We have now passed your article onto the PLOS Production Department who will complete the rest of the pre-publication process. All authors will receive a confirmation email upon publication.

The corresponding author will soon be receiving a typeset proof for review, to ensure errors have not been introduced during production. Please review the PDF proof of your manuscript carefully, as this is the last chance to correct any scientific or type-setting errors. Please note that major changes, or those which affect the scientific understanding of the work, will likely cause delays to the publication date of your manuscript. Note: Proofs for Front Matter articles (Pearls, Reviews, Opinions, etc...) are generated on a different schedule and may not be made available as quickly.

Soon after your final files are uploaded, the early version of your manuscript, if you opted to have an early version of your article, will be published online. The date of the early version will be your article's publication date. The final article will be published to the same URL, and all versions of the paper will be accessible to readers.

For Research Articles, you will receive an invoice from PLOS for your publication fee after your manuscript has reached the completed accept phase. If you receive an email requesting payment before acceptance or for any other service, this may be a phishing scheme. Learn how to identify phishing emails and protect your accounts at https://explore.plos.org/phishing.

Thank you again for supporting open-access publishing; we are looking forward to publishing your work in PLOS Pathogens.

Best regards,

Sumita Bhaduri-McIntosh

Editor-in-Chief

PLOS Pathogens

orcid.org/0000-0003-2946-9497

Michael Malim

Editor-in-Chief

PLOS Pathogens

orcid.org/0000-0002-7699-2064
